# Structural Modeling and *in planta* Complementation Studies Link Mutated Residues of the *Medicago truncatula* Nitrate Transporter NPF1.7 to Functionality in Root Nodules

**DOI:** 10.3389/fpls.2021.685334

**Published:** 2021-07-01

**Authors:** Yao-Chuan Yu, Rebecca Dickstein, Antonella Longo

**Affiliations:** Department of Biological Sciences, BioDiscovery Institute, University of North Texas, Denton, TX, United States

**Keywords:** *Medicago truncatula*, NPF transporters, NIP/LATD, symbiotic nitrogen fixation, nitrate signaling, root architecture analysis

## Abstract

Symbiotic nitrogen fixation is a complex and regulated process that takes place in root nodules of legumes and allows legumes to grow in soils that lack nitrogen. Nitrogen is mostly acquired from the soil as nitrate and its level in the soil affects nodulation and nitrogen fixation. The mechanism(s) by which legumes modulate nitrate uptake to regulate nodule symbiosis remain unclear. In *Medicago truncatula*, the MtNPF1.7 transporter has been shown to control nodulation, symbiosis, and root architecture. MtNPF1.7 belongs to the nitrate/peptide transporter family and is a symporter with nitrate transport driven by proton(s). In this study we combined *in silico* structural predictions with *in planta* complementation of the severely defective *mtnip-1* mutant plants to understand the role of a series of distinct amino acids in the transporter’s function. Our results support hypotheses about the functional importance of the ExxE(R/K) motif including an essential role for the first glutamic acid of the motif in proton(s) and possibly substrate transport. Results reveal that Motif A, a motif conserved among major facilitator transport (MFS) proteins, is essential for function. We hypothesize that it participates in intradomain packing of transmembrane helices and stabilizing one conformation during transport. Our results also question the existence of a putative TMH4-TMH10 salt bridge. These results are discussed in the context of potential nutrient transport functions for MtNPF1.7. Our findings add to the knowledge of the mechanism of alternative conformational changes as well as symport transport in NPFs and enhance our knowledge of the mechanisms for nitrate signaling.

## Introduction

Nitrate is an important nutrient for plant growth and development as well as a signal regulator for metabolic and developmental processes including nitrogen and carbon metabolism and root development (Bouguyon et al., 2012). In legumes, nitrate at high concentrations is a negative regulator of nodulation, while at lower concentrations, it appears to stimulate nodulation. Its effect may be at least partially mediated by the interaction with phytohormones such as auxin (Ferguson and Mathesius, 2014; van Noorden et al., 2016). Nitrate has been proposed to participate in symbiotic nitrogen fixation (SNF) as an alternative electron sink (Horchani et al., 2011).

In the model legume *Medicago truncatula*, NPF1.7, a transporter belonging to the low-affinity NRT1/PTR transporter family (NPF), plays an essential role in meristem development of nitrogen-fixing nodules, lateral and primary roots (Yendrek et al., 2010; Bagchi et al., 2012; Salehin et al., 2013). This transporter was previously called NIP/LATD (for Numerous Infections and Polyphenolics/Lateral root-organ Defective). The phenotypes for plants with defects in *MtNPF1.7*, the *mtnip-1* and *mtlatd* mutants described in Veereshlingam et al. (2004) and in Bright et al. (2005), were profoundly defective. The weaker *mtnip-3* allele (Teillet et al., 2008) also has striking phenotypic defects in roots and nodules. The three mutants have distinct point mutations (Yendrek et al., 2010): the *mtlatd* allele has a premature stop mutation, W341stop, while the *mtnip-1* and *mtnip-3* alleles harbor missense mutations, A497V and E171K, respectively. We continue to refer to the mutants by their original names, *mtnip-1* and *mtnip-3*, here.

Although NPFs have evolved to transport a variety of substrates, MtNPF1.7 has only been shown to transport nitrate at low concentrations (Bagchi et al., 2012; Salehin et al., 2013) and is speculated to transport a phytohormone (Harris and Dickstein, 2010; Zhang et al., 2014), possibly functioning as a N sensor (Gojon et al., 2011). MtNPF1.7, like most NPFs, is a symporter that utilizes a proton-driven force to transport nitrate (Bagchi et al., 2012). Understanding of the transport mechanisms in NPFs is mostly based on the available crystal structures for one plant and for several bacterial proton-coupled oligopeptide transporters (POTs) (Supplementary Table 1 and references therein) and the alternating-access mechanism first proposed by Jardetzky (1966). In this model, active transport in membrane transporters is based on two stable conformations, the inward-open (I_o_) and the outward-open (O_o_) conformations that offer the substrate alternating-access to the cytoplasm and the extracellular space, respectively. Since then the model has evolved to include a better understanding of the conformational changes of the transporters that are coupled to the transport process (Newstead, 2015; Zhang et al., 2015).

Analysis of the *Arabidopsis thaliana* dual affinity nitrate transporter AtNPF6.3 structure reveals a canonical Major Facilitator Superfamily (MFS) fold with 12 trans-membrane helices (TMHs) with a central linker region between TMH6 and TMH7 that partially folds into a short amphipathic α-helix named lateral helix (Parker and Newstead, 2014; Sun et al., 2014) (Supplementary Figure 1). The N- and C-terminal six-helix bundles form a ‘V’ shaped transporter, related by a pseudo two-fold symmetry axis running perpendicular to the membrane plane. The structure was captured with the transporter in the I_o_ state with the “V” open towards the cytoplasmic side of the membrane. The E_1_xxE_2_(R/K) motif, located on TMH1, includes residues Glu41 (E_1_), Glu44 (E_2_), and Arg45 (R/K) (Supplementary Figure 1A). The motif is highly conserved in most NPF subfamilies (Longo et al., 2018), in bacterial POTs (Newstead, 2015), and in diatom NPFs (Santin et al., 2021). Mutagenesis studies have shown that the chargeable residues in this motif play essential roles including proton(s) binding and substrate-transport coupling. Other residues have been implicated in substrate-transport coupling including two residues with opposite charges located on TMH4 and TMH10 that have been proposed to form a salt bridge in the O_o_ conformation and to orchestrate dynamic movements during the transport cycle in POTs and NPFs (Parker and Newstead, 2014; Fowler et al., 2015; Newstead, 2015).

All available crystal structures of plant NPFs and bacterial POTs were solved in the I_o_ conformation (Supplementary Table 1) and therefore there are no available structures for these transporters in the O_o_ conformation. However, the structure of a transporter belonging to MFS, *Escherichia coli* YajR, has been solved in the O_o_ conformation (Jiang et al., 2013). YajR is folded in the canonical 12-helix MFS topology, with two domains containing six TMHs each and connected by a 30-residue linker, partially folded in α-helix (Supplementary Figure 2). The connecting helix is similar to the lateral helix in AtNPF6.3, but in YajR the helix is separated from TMH7 by only one amino acid, while in AtNPF6.3 the lateral helix and TMH7 are separated by a long unstructured loop of 65 amino acids (Supplementary Figures 1, 2). Unlike AtNPF6.3 and other MFS structures, YajR contains a highly negatively charged domain with a ferredoxin-like fold termed YAM domain. The YAM domain is located in the cytoplasmic side of the membrane at the carboxyl terminus of the protein and it has been suggested to have a regulatory role instead of a structural one. A central cavity forms between the N- and C-domains in the membrane portion of the protein and, differently from AtNPF6.3, it is closed towards the cytoplasm and open towards the extracellular space. YajR does not contain an ExxE(R/K) motif, with His225 (TMH7) and Glu320 (TMH10) being considered as possible candidates for protonation (Supplementary Figure 2). The substrate transported by YajR is not known. The YajR structure can be used for homology modeling of NPFs and other MFSs in the O_o_ conformation (Date et al., 2016).

This study is a structural and functional analysis to understand which residues are necessary for functionality of MtNPF1.7 including the ExxE(R/K) motif residues and the residues forming the TMH4-TMH10 salt bridge. We also address the role of two amino acids, Ala497 and Glu171, that in the *mtnip-1* and *mtnip-3* mutants, respectively, cause compromised legume-Rhizobium symbiosis and altered lateral root growth (Veereshlingam et al., 2004; Bright et al., 2005; Teillet et al., 2008; Yendrek et al., 2010). Our work is based on homology-modeling of MtNPF1.7 built on the available crystal structures described above followed by *in planta* experiments that test our hypothesis by complementing a defective *Medicago* line. Our studies support and add to the proposed working model for transporter activity of proton-nitrate symporters.

## Materials and Methods

### Sequence Alignments

Multiple sequence alignments were performed using the Clustal Omega program via the Web Services interface at the European Bioinformatics Institute (EMBL-EBI) (Sievers et al., 2011; McWilliam et al., 2013) using a collection of plant NPF amino acid sequences as in Longo et al. (2018). Logos were created using WebLogo3^1^ (Crooks et al., 2004).

### Structural Modeling

Structural homology-modeling was performed using the automated SWISS-MODEL ExPASy webserver^2^ (Arnold et al., 2006; Waterhouse et al., 2018). A structural model of MtNPF1.7 in the inward-open conformation was obtained using the AtNPF6.3 crystal structure (pdb: 4oh3, chain B) (Sun et al., 2014) as template. Structural models of MtNPF1.7 and AtNPF6.3 in the outward-open conformation (O_o_) were obtained using the crystallographic coordinates of the *E. coli* YajR transporter (pdb: 3wdo) (Jiang et al., 2013) as template. Spatial arrangement of the structures in lipid bilayers was calculated using the Orientations of Proteins in Membranes (OPM) server^3^ (Lomize et al., 2006, 2012). Structures were visualized with the molecular graphics software PyMOL (The PyMOL Molecular Graphics System, Schrödinger, LLC).

### Plant Material and Growth Conditions

In this study we used *M. truncatula* plants of the genotype Jemalong A17 as the wild types (WTs). The mutant line named *mtnip-1*, obtained via ethyl methane sulfonate (EMS) induced mutagenesis from the A17 genetic background (Veereshlingam et al., 2004; Yendrek et al., 2010) and containing the A497V mutation, was used for complementation studies. WT and *mtnip-1* plants were grown under 16/8 hr light/dark at 22°C, as previously described (Veereshlingam et al., 2004). For plant hairy root transformation (Boisson-Dernier et al., 2001), plant seedlings were grown in Fahräeus medium (containing 1.5 mM nitrate) under 16/8 h light/dark at 20°C for 7 days, followed by 25°C for 14 days.

### *MtNPF1.7* Mutagenesis

The full-length open reading frames of WT *MtNPF1.7* and mutants *mtnip-1* and *mtnip-3* were obtained from pcDNA3.1-nipc, pMS217, and pMS219, respectively (Bagchi et al., 2012), by restriction digestion with NcoI and SalI and subcloned into pGEM^®^-T Easy (Promega), creating pYCY200, pYCY201, and pYCY202, respectively. These plasmids were used for site-directed mutagenesis to obtain plasmids pYCY203 to pYCY220 (Supplementary Table 2). Two different methods were used to obtain mutants of *MtNPF1.7*: a first group of mutants were obtained using the Phusion Site-Directed Mutagenesis Kit (ThermoFisher Scientific), a second group of mutants were obtained using the Q5^®^ Site-Directed Mutagenesis Kit (New England Biolabs Inc.).

The expression construct *MtNPF1.7* (p*AtEF1a*-*MtNPF1.7*-*eGFP*) was obtained from pMS210 (Bagchi et al., 2012) and recombined into pGEM^®^-T Easy through KpnI and XhoI sites with the Gibson assembly method (NEBuilder^®^HiFi DNA Assembly, New England BioLabs, Inc.) to obtain pYCY300. Full-length site-directed mutants (pYCY203 to pYCY220) were subcloned into pYCY300 through NcoI and NheI sites by substituting the *MtNPF1.7* fragment, generating plasmids pYCY301 to pYCY320 (Supplementary Table 2). p*AtEF1a*-*mtnpf1.7*mutants-*eGFP* (pYCY301 to pYCY320) were subcloned into the KpnI and XhoI sites of the binary vector pMU06 (generous gift of Wei Liu and Michael Udvardi, Noble Research Institute, Ardmore, OK) to replace the GUS gene, forming plasmids pYCY400 to pYCY420 (Supplementary Table 2). Plasmids pYCY400 to pYCY420 were transformed into *Agrobacterium rhizogenes* ARqua1 strain (Quandt et al., 1993) via electroporation.

Primers used for mutagenesis are listed in Supplementary Table 3, primers used for cloning and sequencing in Supplementary Table 4. Mutations were confirmed by sequencing (Eurofins Genomics, Louisville, KY, United States).

### Complementation Studies

For complementation analysis, the wild-type A17 and *mtnip-1* seeds were surface sterilized and germinated. The 1-day-old seedlings were used for plant hairy root transformation (Boisson-Dernier et al., 2001) by inoculating with *A. rhizogenes* ARqua1 that had been transformed with pMU06-based plasmids (pYCY400 to pYCY420) that contained either the wild type *MtNPF1.7*-*eGFP* or its mutated variants. Proteins were expressed under the control of the *A. thaliana* EF1*a* gene promoter (pAtEF1*a*) which results in constitutive expression (Auriac and Timmers, 2007). The transgenic plants were selected by observing DsRed fluorescence in transformed roots under a dissecting microscope, Leica MZ10F (Leica Microsystems Inc., Buffalo Grove, IL, United States), and then excising the non-transgenic roots. The selected transgenic plants were grown in aeroponic chambers supplied with 14 L of Lullien medium (Lullien et al., 1987) containing 5 mM ammonium nitrate for 3 days, followed by 3 days of nitrate deprivation, and finally inoculated with *Sinorhizobium meliloti* strain ABS7 (Penmetsa and Cook, 1997). Overnight grown ABS7 culture were washed and resuspended in 20 mL H_2_O before adding to the aeroponic chamber for inoculation. Plants were harvested at 15 days post-inoculation (dpi) and evaluated for complementation of their nodulation and lateral root phenotypes. These were analyzed and documented using a Leica MZ10F dissecting microscope. The picture of root and nodule phenotype in each mutant is representative from at least 15 transgenic plants in each experiment, and the phenotype is replicable from at least two biological experimental repeats. Root nodules were assessed visually on a scale of 0-2, with 0 representing no complementation and 2 representing full complementation. In addition, we used the nodule images to measure their surface areas using Fiji software, derived from ImageJ (Schindelin et al., 2012). There was a good correlation between visual assessment and nodule area, except in the case of the A89G_A497V mutation, as mentioned in the results. The length of shoots and roots and the fresh weight of plants were measured at 16 dpi. Data were analyzed by ANOVA and the *post hoc* Tukey’s HSD (honestly significant difference) test (*P* < 0.05).

### Accession Numbers

Sequences for MtNPF1.7, AtNPF6.3, and *E. coli* YajR can be found at Genbank accession numbers GQ401665, NM_101083, and NC_000913, respectively.

## Results

### Homology Modeling of the *MtNPF1.7* Nitrate Transporter

We used the atomic coordinates from two transporters, AtNPF6.3 and *E. coli* YajR, as templates for homology modeling of MtNPF1.7 in two different conformations of the alternating-access cycle, I_o_ and O_o_, respectively. This is because a crystal structure for the MtNPF1.7 is not available and AtNPF6.3 and YajR are the closest MtNPF1.7 homologs with solved structures in the respective conformations.

**FIGURE 1:**
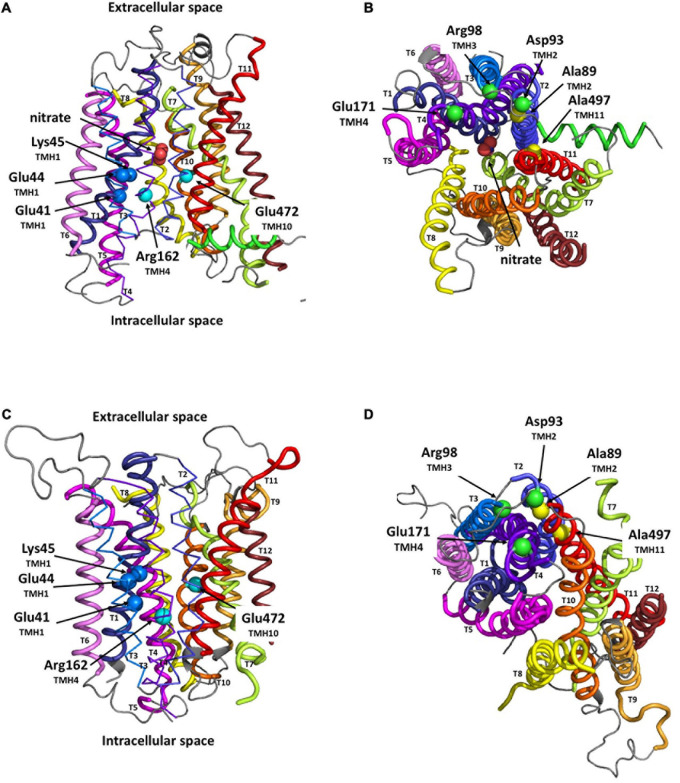
Structural models of MtNPF1.7 in two different conformations. Loop rendering of the peptide backbone of the *in silico* models of MtNPF1.7. Templates for homology modeling were the crystal structures of AtNPF6.3 for the inward-open (I_o_) conformation (pdb: 4oh3) (Sun et al., 2014) in panels **(A,B)** and of *E. coli* YajR for the outward-open (O_o_) conformation (pdb: 3wdo) (Jiang et al., 2013) in panels **(C,D)**. Important residues are shown as spheres corresponding to their Cα atoms. **(A,C)** MtNPF1.7 models as viewed from the membrane with the ExxE(R/K) motif residues Glu41, Glu44, and Lys45 as dark blue spheres, and two residues, Arg162 and Glu472 predicted to form a TMH4-TMH10 salt bridge in the O_o_ conformation as cyan spheres. **(B,D)** 90° rotation of panels **(A,C)** for a view from the cytoplasmic side of the membrane with residues involved in the salt-bridge triad, Asp93, Arg98, and Glu171, as green spheres, and residues important for packing in the O_o_ conformation, Ala89 and Ala497, as yellow spheres.

The I_o_ model of MtNPF1.7, obtained using the structural data from AtNPF6.3 (Sun et al., 2014) as template, shows the protein folded in the canonical MFS fold of 12 TMHs organized into two bundles of six TMHs each, TMH1-6 and TMH7-12 (Figure 1A). A short inter-domain connecting region between TMH6 and TMH7 is partially folded into a lateral helix located in the cytoplasmic space parallel to the lipid membrane. A partially hydrophilic transporter channel is formed between the two bundles and provides the pathway for transporting proton(s) and nitrate. Residues from the conserved ExxE(R/K) motif on TMH1 have their side chains exposed to the cytosolic solvent in the transporter tunnel. In this I_o_ conformation, the transporter opens towards the cytoplasmic side with the putative binding site for nitrate located near the extracellular side and tightly sealed by the packing of TMH1-TMH2 against TMH7-TMH8 forming the extracellular gate (Figure 1B).

In the MtNPF1.7 model in the O_o_ conformation, obtained using the YajR transporter data (Jiang et al., 2013), the protein is open towards the extracellular side (Figures 1C,D). In this conformation, TMH1-TMH2 have moved far from TMH7-TMH8 on the extracellular side, while TMH2, TMH3, TMH4 and TMH11 are packed on the cytoplasmic side forming the intracellular gate (Figures 1C,D).

Our models of MtNPF1.7 in the two conformations provide a structural framework to our investigation of the role of specific residues and their contribution to the mechanism of substrate translocation and recognition. We used the positions of the modeled structural features to predict intramolecular sites to target for mutagenesis and then to test for function.

### *In planta* Complementation Studies of the *mtnip-1* Mutant

*In planta* complementation studies were performed to investigate specific MtNPF1.7 amino acids known or predicted to play an important role in the alternating-access mechanism of NPFs, POTs, or other MFS transporters. For this purpose, we used a *M. truncatula* plant line, *mtnip-1*, with a well-defined altered phenotype caused by a missense mutation, A497V, in the *MtNPF1.7* gene. When grown in symbiotic conditions, *mtnip-1* shows extreme root system defects, with altered nodulation phenotype and aberrant root architecture (Veereshlingam et al., 2004; Yendrek et al., 2010; Supplementary Figure 3A). *Mtnip-1* plants develop nodules that initiate rhizobial invasion but fail to release rhizobia from infection threads, accumulate a brown pigment due to polyphenolics, a sign of host defense, and lack the typical pink color, associated with functional leghemoglobin protein. *Mtnip-1* plants have primary roots that are shorter than wild-type and stunted lateral roots. The wild-type phenotype including plant-rhizobia symbiotic interaction and root architecture can be restored by complementation when *mtnip-1* plants are transformed with a vector harboring either a genomic (Yendrek et al., 2010) or a cDNA version (Bagchi et al., 2012) of the *MtNPF1.7* gene (Supplementary Figure 3A).

Based on these observations, we decided to use complementation of the defective *mtnip-1* plants to test a series of *MtNPF1.7* mutants (Table 1) with the expectation that mutating residues critical to MtNPF1.7 functionality will result in no complementation while mutating not critical residues will result in complementation and a rescued phenotype (Supplementary Figure 3B).

**TABLE 1:** List of mutants used for *in planta* complementation studies, their postulated role, and complementation results with 0 = no complementation, 1 = partial complementation, 2 = full complementation.

**Mutation**	**Postulated Role**	**Complementation**
E41A	Proton transport	0
E44A	Proton transport	2
K45A	Proton transport	2
K45R	Proton transport	2
R162A	Salt bridge (TMH4-TMH10)	2
E472A	Salt bridge (TMH4-TMH10)	2
R162E/E472R	Salt bridge (TMH4-TMH10)	0
E171K (*mtnip-3*)	Salt bridge triad	1
E171A	Salt bridge triad	2
D93A	Salt bridge triad	2
R98A	Salt bridge triad	2
D93A/E171A	Salt bridge triad	0
R98A/E171A	Salt bridge triad	2
D93R/E171K	Salt bridge triad	0
R98D/E171K	Salt bridge triad	2
D93R/R98D/E171K	Salt bridge triad	2
A497V (*mtnip-1*)	Helix-helix packing	0
A89G/A497V	Helix-helix packing	2

### *In planta* Complementation Studies Tests the Requirement for the ExxE(R/K) Motif in MtNPF1.7’s Functionality

A conserved motif on TMH1 called the E_1_xxE_2_(R/K) motif has been implicated in proton binding and transport in plant NPFs and bacterial POTs. Residues in the motif may also have a role in driving the conformational changes needed to alternate between O_o_ and I_o_ conformations. Mutagenesis studies of the chargeable amino acids in the motif have been performed in AtNPF6.3 (Ho and Frommer, 2014; Parker and Newstead, 2014; Sun et al., 2014) and AtNPF2.11 (Jørgensen et al., 2015) as well as in the bacterial oligopeptide transporters PepT_*St*_ (Solcan et al., 2012), PepT_*So*_ (Fowler et al., 2015), and GkPOT (Doki et al., 2013) (Supplementary Table 5). Mutating any of the two glutamic acids or the arginine/lysine resulted in the loss of proton-driven transport activity in heterologous systems like oocytes and liposomes (Supplementary Table 5). However, counterflow activity which is the ability of a transporter to mobilize a substrate from a higher to a lower concentration without an energy gradient, was preserved for these mutants in PepT_*St*_ (Solcan et al., 2012). In a recently proposed model, the two glutamic acids are sites for protonation, while the arginine can swap conformation and alternatively form an R-E_2_ or R-E_1_ salt bridge (Aduri et al., 2015). Other studies on the ExxE(R/K) motif reveal discrepancies with the model. For instance, while mutating these residues abolishes the proton-driven transport activity, the counterflow activity is maintained in PepT_*St*_ (Solcan et al., 2012), but it is lost in GkPOT (Doki et al., 2013). Interestingly, in the bacterial POTs YbgH and YjdL, the lone glutamic acid in the first position of the ExxE(R/K) motif is essential for proton-driven transport activity (Zhao et al., 2014; Aduri et al., 2015).

**FIGURE 2:**
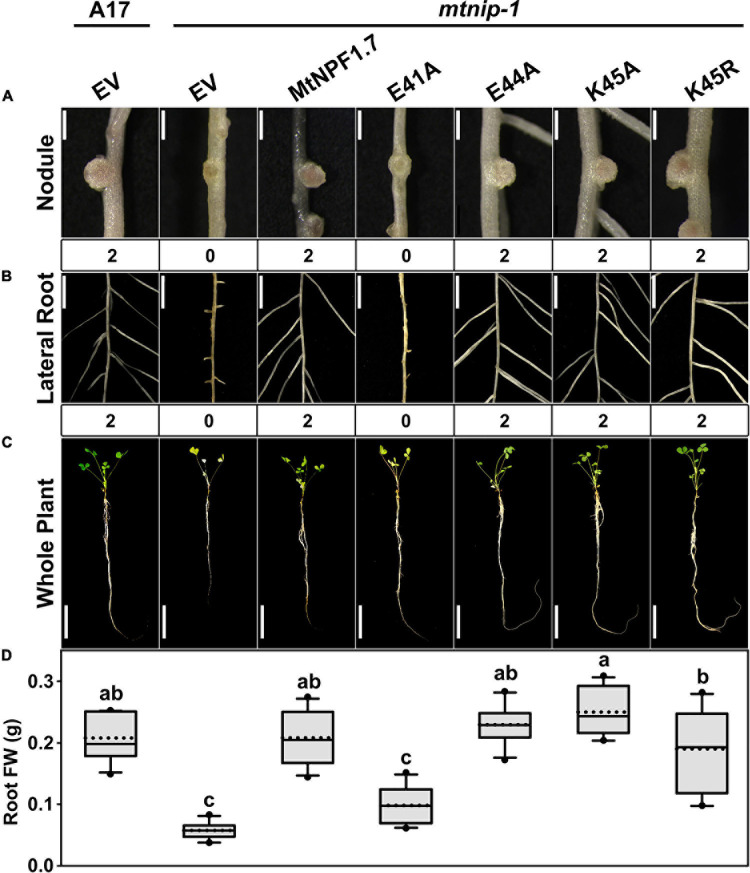
Results of *in planta* complementation for the ExxE(R/K) motif mutants. Images showing **(A)** the nodules, **(B)** the lateral roots and **(C)** the whole plants of (from left to right) A17 plant roots transformed with the pMU06 empty vector (EV) and *mtnip-1* plant transformed with the EV, MtNPF1.7_wt (pYCY400), _E41A (pYCY403), _E44A (pYCY404), _K45A (pYCY405), or _K45R (pYCY406). Nodules and lateral roots were evaluated by complementation levels of defective phenotypes shown below each image: 0 = no complementation, 1 = partial complementation, 2 = full complementation. **(A)** Bars = 1 mm. **(B)** Bars = 5 mm. **(C)** Bars = 5 cm. **(D)** Plots of root fresh weight from ten independent transgenic plants (*n* = 10). Data are shown as box plots. The lower and upper boundaries of the box indicate the 25th and 75th percentiles, respectively, with the median and mean marked as solid and dotted lines, respectively. The ends of lower and upper whiskers indicate the minimum and maximum values. Outliers are shown as dots. The letters above each box indicate the significant differences analyzed by ANOVA and the *post hoc* Tukey’s HSD (honestly significant difference) test (*P* < 0.05). Only the E41A mutant failed to complement the *mtnip-1* phenotype, while E44A, K45A and K45R restored the wild type phenotype.

In MtNPF1.7, the ExxE(R/K) motif corresponds to residues E41-E44-K45 and in the structural model in the I_o_ conformation, their side chains are positioned to face the central cavity and exposed to interaction with the solvent (Figure 1A). The motif is predicted to play a role in proton transport and coupling as in other plant NPFs and bacterial POTs. To test this prediction, we mutated the chargeable amino acids in the motif to alanines and obtained the E41A, E44A, K45A mutants (Table 1). Plasmids carrying the mutated transporters under the constitutive p*AtEF1a* promoter were tested in *mtnip-1* plants for complementation in root transformation experiments. When *mtnip-1* plants were transformed with a plasmid carrying the mutated MtNPF1.7_E41A, the plants showed defective nodulation and root architecture indicating that the mutated protein could not complement the *mtnip-1* phenotype (Figure 2 and Supplementary Figure 4). In contrast, *mtnip-1* plants transformed with the MtNPF1.7_E44A mutant had a restored phenotype with pink nodules and normal lateral roots. We also found that *mtnip-1* plants transformed with plasmids carrying either the mutated MtNPF1.7_K45A or MtNPF1.7_K45R complemented the *mtnip-1* phenotypes (Figure 2 and Supplementary Figure 4).

### *In silico* Analysis Combined With *in planta* Complementation Studies to Assess the Putative TMH4-TMH10 Salt Bridge Formation in MtNPF1.7

It has been proposed that a salt bridge could form in the O_o_ conformation of bacterial POTs between two residues with opposite charges located on TMH4 and TMH10 (Solcan et al., 2012; Fowler et al., 2015; Newstead, 2015). The TMH4-TMH10 salt bridge facilitates packing of the helices stabilizing the O_o_ conformation. In the bacterial POTs, PepT_*St*_ and GkPOT, mutating the corresponding residues on TMH4 (Lys126 and Lys136, respectively) and on TMH10 (Glu400 and Glu310, respectively) abolished the proton-driven transport in proteoliposomes, but the peptide-driven counterflow uptake was retained for the TMH4 mutants (Solcan et al., 2012; Doki et al., 2013). Similarly, in the plant AtNPF6.3, when either Lys164 (TMH4) or Glu476 (TMH10) were mutated, the proton-driven nitrate uptake tested in oocytes, was dramatically diminished for both mutants with only the Lys164A mutant retaining the counterflow activity (Ho and Frommer, 2014; Parker and Newstead, 2014; Sun et al., 2014). These results could indicate that the glutamic acid on TMH10 may have an additional role in substrate transport and therefore it is strictly required for transport. It is important to notice that the TMH4-TMH10 salt bridge has not been experimentally observed as all POTs and NPFs crystal structures have been solved in the I_o_ or occluded conformations but not in the O_o_ conformation when this salt bridge is predicted to form (Supplementary Table 1).

We sought to test whether the residues that can form the putative TMH4-TMH10 salt bridge are essential in MtNPF1.7. Based on sequence alignment, we predict that in MtNPF1.7 the residues forming the putative salt bridge are Arg162 (TMH4) and Glu472 (TMH10) (Figures 1A,C). In the I_o_ conformation model, based on the AtNPF6.3 structure, the distance between the closest atoms of Arg162 and Glu472 is 8.2 Å, insufficient to form a salt bridge in this conformation which is expected. The two amino acids are predicted to lie within a hydrogen bonding distance in the O_o_ conformation, enough for interaction of the two amino acids with opposite charge. However, in our model of MtNPF1.7 in the O_o_ conformation, based on the YajR structure, the distance between the two amino acids, Arg162 and Glu472, increases to 14 Å (Figure 1C). This observation is surprising as the formation of a salt bridge in the O_o_ conformation is at the basis of the proposed alternating-access mechanism (Fowler et al., 2015; Newstead, 2015).

It is important to notice that a salt bridge between TMH4 and TMH10 was not observed in the crystal structure of YajR in the O_o_ conformation as the corresponding residues in TMH4 and TMH10 are Ala117 and Phe316, respectively. A negatively chargeable residue, Glu320, is located one helix turn from Phe316 and can form hydrogen bonds with His225 (TMH7). There are two positively chargeable residues in the transporter access cavity, Arg24 (TMH1) and Arg108 (TMH4), but in the crystal structure they are too far to be able to form a salt bridge with Glu320. These observations may also indicate that YajR could use a different mechanism in the alternate cycle or reaches a different conformation that does not require the formation of a salt bridge between TMH4 and TMH10.

**FIGURE 3:**
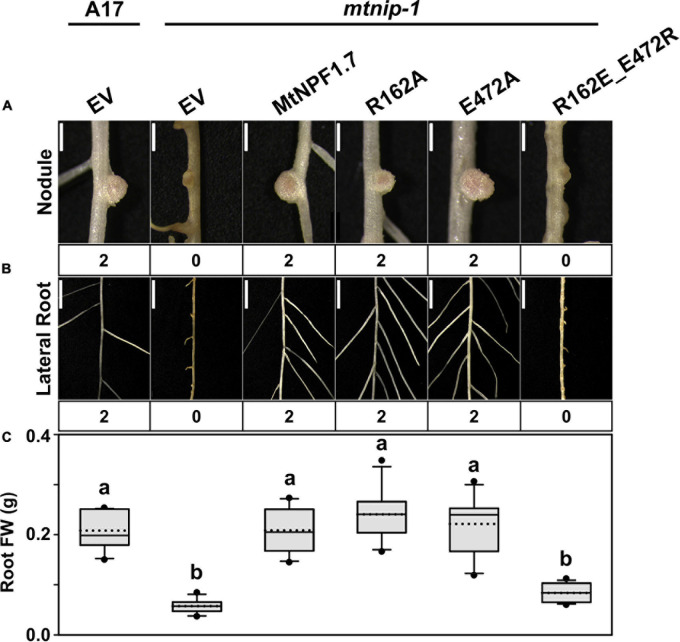
Results of *in planta* complementation with the TMH4-10 salt bridge mutants. Images showing **(A)** the nodules and **(B)** the lateral roots of (from left to right) A17 plant roots transformed with the pMU06 empty vector (EV) and *mtnip-1* plant roots transformed with EV, MtNPF1.7_wt (pYCY400), _R162A (pYCY407), _E472A (pYCY408), and _R162E-E472R (pYCY411). Nodules and lateral roots were evaluated by complementation levels shown below each image as described in Figure 2. **(A)** Bars = 1 mm. **(B)** Bars = 5mm. **(C)** Plot of root fresh weight from ten independent transgenic plants. See Figure 2D for statistical details. Plants transformed with the MtNPF1.7_R162A and _E472A mutants have a restored phenotype. The double mutant, MtNPF1.7_R162E-E472R, designed to swap the charges of the putative TMH4-TMH10 salt bridge does not rescue the mutated phenotype.

To experimentally assess the role Arg162 and Glu472 in MtNPF1.7, we created the Arg162Ala and the Glu472Ala mutants as well as a double mutant with swapped charges, R162E-E472R. The mutants were tested in planta separately (Table 1). Both single mutants complemented the *mtnip-1* phenotype, while the double mutant with swapped charges did not rescue the phenotype (Figure 3 and Supplementary Figures 4B, 5D-F, 6A). Because swapping the charge of both residues is not enough to restore the phenotype, we can speculate that the salt bridge does not form between these two residues. This is consistent with our observation that in the structural model of MtNPF1.7 in the O_o_ conformation the two residues are too far from each other (Figure 1C), suggesting that a salt bridge cannot form in this conformation.

### *In silico* Analysis and *in planta* Complementation of the *mtnip-3* Mutation (E171K) to Probe a Potential Salt Bridge Triad on the Cytoplasmic Side of *MtNPF1.7*

The *mtnip-3* mutant shows polyphenol accumulation in the nodules and significant reduction of N fixation and has mild defects in lateral root elongation and architecture (Teillet et al., 2008; Yendrek et al., 2010; Salehin et al., 2013). The *mtnip-3* phenotype is caused by a missense mutant, E171K. So far no hypothesis has been proposed for a role of this residue in MtNPF1.7 or in other NPFs. Therefore, we used *in silico* analysis of the MtNPF1.7 models (Figure 1) to investigate how the E171K mutation may compromise MtNPF1.7 function.

**FIGURE 4:**
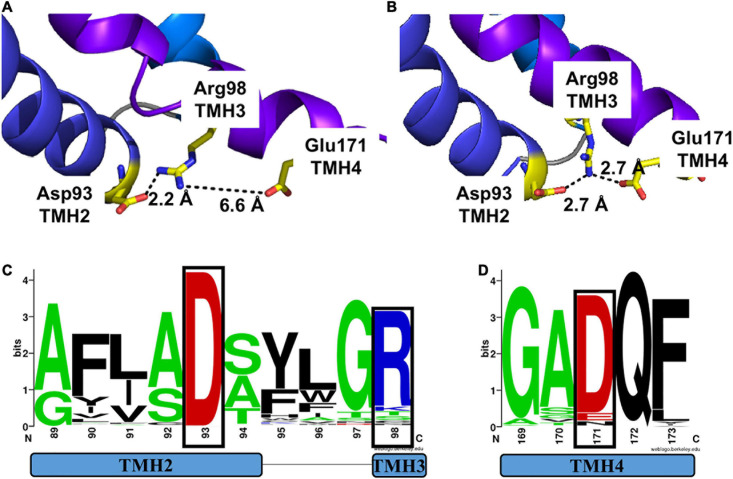
Motif A in two different conformations of MtNPF1.7 and its conservation in plant NPFs. Comparison of the salt-bridge triad in two MtNPF1.7 structural models based on the crystal structure of panel **(A)** the AtNPF6.3 transporter solved in the I_o_ conformation (pdb: 4oh3) (Sun et al., 2014) or **(B)** the *E. coli* YajR transporter solved in the O_o_ conformation (pdb: 3wdo) (Jiang et al., 2013). **(A)** In the I_o_ conformation, Arg98 forms a salt bridge with Asp93, but Glu171 is too distant (6.6 Å). **(B)** In the O_o_ conformation Arg98 can form a salt bridge with both Asp93 and Glu171, thanks to the close packing of the TMHs on the cytoplasmic side. **(C)** Sequence logo of Motif A showing conservation of residues in position +5 (Asp93 in MtNPF1.7) in TMH2 and +10 (Arg98 in MtNPF1.7) in TMH3 (residues in a black box). **(D)** Sequence logo showing conservation of Glu171 in TMH4. Logos obtained using a collection of 2,380 sequences from plant NPFs (Longo et al., 2018). Numbering based on the MtNPF1.7 sequence. Color scheme: short side chains amino acids (Gly, Ala, Ser, and Thr) are in green; basic (Arg, Lys, His) in blue; acidic (Asp and Glu) in red; others in black.

We first analyzed the structural models for MtNPF1.7 in two conformations. In the MtNPF1.7 model in the I_o_ conformation, Glu171 is located at the C-terminal end of TMH4 and is at 6.6 Å from Arg98 at the C-terminal end of TMH3 (Figures 1B, 4A). In this conformation, an amino group from Arg98 is at 2.2 Å from the carboxyl group of Asp93 indicating participation in a salt bridge. In the MtNPF1.7 model in the O_o_ conformation, the three residues are closer to each other and the carboxyl groups of both Asp93 and Glu171 are in the distance range for a salt bridge formation with the amino groups of Arg98 (Figures 1D, 4B).

To confirm our observations on the MtNPF1.7 models, we examined the crystal structures used as templates for homology modeling. Sequence alignment between MtNPF1.7 and AtNPF6.3 shows that Glu171 is conserved in AtNPF6.3 where it corresponds to residue Asp173. In the crystal structure, Arg98 forms a salt bridge with Asp93 located at the N-terminal end of TMH2 but Asp173 is not close enough for a salt bridge (Supplementary Figure 7A), similar to the model for MtNPF1.7 in this conformation. Because there are three available crystal structures for AtNPF6.3 with the protein crystallized as a dimer in all structures (Parker and Newstead, 2014; Sun et al., 2014), we analyzed the distance between the three amino acids from both chains in each structure. We observed that the distance between residues is variable (Table 2). In the structures, that were all solved in the I_o_ conformation, the distance between Asp93-Arg98 is in the 2.2 and 4.3 Å range, while the distance between Arg98 and Asp173 is in the 4.7 to 7.6 Å range. Because the distance for a proper bond acceptor-donor pair in a salt bridge is 2.7 to 3.3Å, the distance between Asp93 and Arg98 support the formation of a salt bridge in all structures, while the distance between Arg98 and Asp173 is too large for the formation of a salt bond. However, the observed differences in distance between the amino acids in different crystal structures of the same protein reflect the dynamic nature of the salt bridges that form between the three residues in this conformation. This is also reflected in the B-factor values that are higher in the loop regions connecting the TMHs on the cytoplasmic side of the protein than in the membrane imbedded TMHs (not shown).

**TABLE 2:** Distance between residues from Motif A (Asp93 and Arg98) and from TMH4 (Asp173) in the dimers of AtNPF6.3 crystal structures.

**PDB**	**Chain**	**Asp93-Arg98**	**Asp93-Asp173**	**References**
**4oh3**	**A**	2.7 Å	4.7 Å	Sun et al., 2014
**4oh3**	**B**	2.2 Å	7.6 Å	Sun et al., 2014
**5a2n**	**A**	2.5 Å	7.0 Å	Parker and Newstead, 2014
**5a2n**	**B**	4.3 Å	5.1 Å	Parker and Newstead, 2014
**5a2o**	**A**	2.5 Å	6.3 Å	Parker and Newstead, 2014
**5a2o**	**B**	3.2 Å	4.7 Å	Parker and Newstead, 2014

In the absence of a crystal structure solved in the O_o_ conformation for NPFs and POTs, we analyzed the structure of *E. coli* YajR that was solved in this conformation (Jiang et al., 2013). In YajR, Arg77, that corresponds to Arg98 in MtNPF1.7, interacts with the side chains of both Asp73 and Asp126, corresponding to Asp 93 and Glu171 in MtNPF1.7, respectively, to form a charge-relay system of two salt bridges (Supplementary Figure 7B). It was proposed that the formation of the salt bridge triad stabilizes the outward conformation of YajR (Jiang et al., 2013).

Asp93 and Arg98 from MtNPF1.7 belong to a conserved helix-turn-helix motif named Motif A with sequence G_1_xxx**D**_5_xx(x)G**R**_10_ that has been identified among MFS transporters in the cytoplasmic loop between TMH2 and TMH3 (Paulsen and Skurray, 1994; Jessen-Marshall et al., 1995; Paulsen et al., 1996). Sequence alignment of 2,388 plant NPFs reveals that Asp93 (position 5 of the motif) on TMH2 is 100% conserved while Arg98 (position 10 of the motif) on TMH3 is slightly less conserved (86%) (Figure 4C). Glu171 on TMH4 does not belong to a specific motif but a positively chargeable residue in this position is well conserved in NPFs, with most NPFs harboring an aspartic acid (86 %) and some a glutamic acid or an asparagine (Figure 4D).

To our knowledge these residues had not been investigated in NPFs before this study. However, several mutagenesis studies have been performed in POTs and other MFS transporters on residues in analogous positions in their respective structures. Mutating any of the three residues in YajR decreased the thermal stability of the protein and increased occupancy of the inward conformation suggesting a role for this system of salt-bridges in stabilizing the O_o_ conformation (Jiang et al., 2013). Two residues from Motif A, Asp79 on TMH2 and Lys84 on TMH3, were mutated in bacterial POT PepT_*So*_ compromising the protein’s ability to transport the AlaAla peptide (Fowler et al., 2015). In the bacterial lactose transporter LacY, the Asp68 mutation resulted in complete loss of transport activity (Jessen-Marshall et al., 1995). Changes to Asp67 in the tetracycline resistance protein, TetA(P), resulted in abolished tetracycline resistance (Kennan et al., 1997). When Asp109 from the proton-coupled folate transporter (PCFT) was mutated, it resulted in loss of activity (Shin et al., 2010; Date et al., 2016; Aluri et al., 2019). Such observations support the hypothesis that Motif A has an important role in stabilizing the O_o_ conformation with the transporters locked into, or preferentially assuming, the I_o_ conformation when Motif A residues are mutated, therefore disrupting the alternating-access mechanism.

These observations led us to hypothesize that the E171K mutation in *mtnip-3* may compromise the formation of the triad salt bridge because of the change in the side chain charge. Since this is a charge reversal mutation with the charge mutated from negative to positive, repulsion between the now like-charged Lys171 and Arg98 likely causes the disruption of the salt bridge between the two residues resulting in an unstable O_o_ conformation. To test the role of the three residues in the protein activity, we mutated them with varying charges to see if we could disrupt the formation of the triad salt bridge and interfere with the protein functionality. We also designed mutants that can reconstitute the salt bridges by charge swapping (Table 1).

**FIGURE 5:**
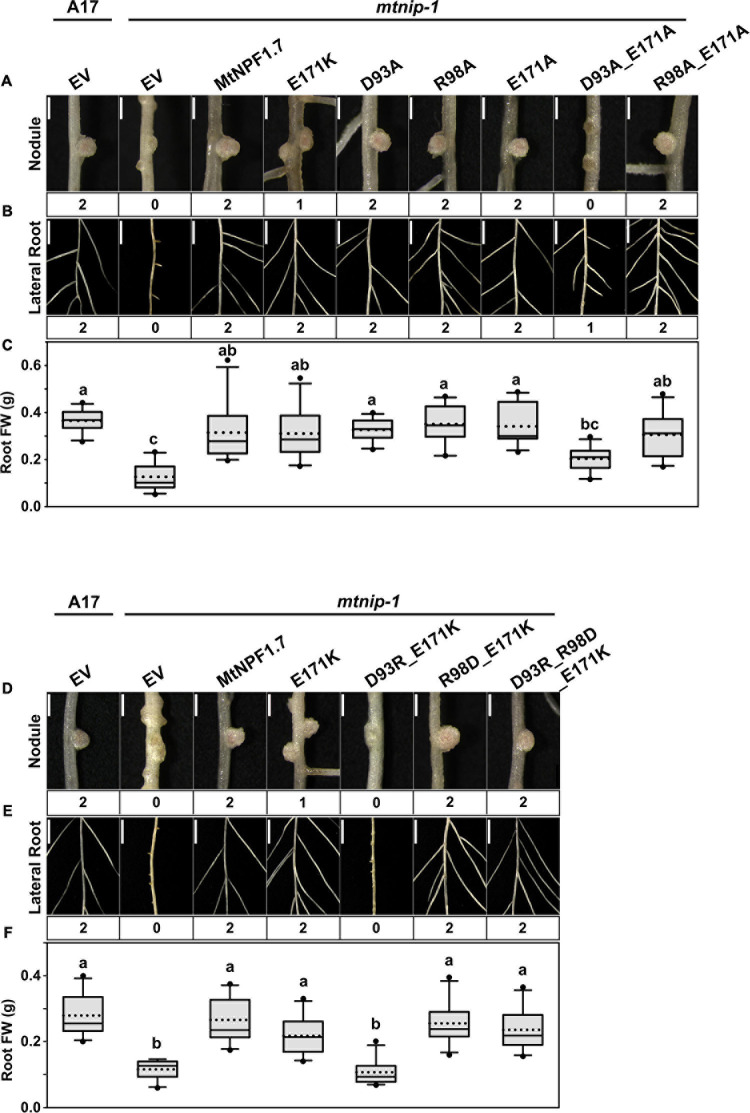
Results of *in planta* complementation for Motif A mutants. Images showing **(A)** the nodules, **(B)** the lateral roots, and **(C)** plots of root fresh weight from ten independent transgenic plants of (from left to right) A17 plant roots transformed with pMU06 empty vector (EV), and *mtnip-1* plant roots transformed with EV, MtNPF1.7_wt (pYCY400), _E171K (pYCY402), _D93A (pYCY412), _R98A (pYCY413), _E171A (pYCY414), _D93A-E171A (pYCY415), and _R98A-E171A (pYCY416). Images showing **(D)** the nodules, **(E)** the lateral roots, **(F)** plots of root fresh weight from ten independent transgenic plants of (from left to right) A17 plant roots transformed with EV, and *mtnip-1* plant roots transformed with EV, MtNPF1.7_wt (pYCY400), _E171K (pYCY402), _D93R-E171K (pYCY417), _R98D-E171K (pYCY418), and _D93R-R98D-E171K (pYCY419). Nodules and lateral roots were evaluated by complementation levels shown below each image as described in Figure 2. **(A,D)** Bars = 1 mm. **(B,E)** Bars = 5 mm. **(C,F)** See Figure 2D for statistical details.

*In planta* complementation was used again to test the mutants. Here we point out that nitrate uptake in *Xenopus laevis* oocytes expressing *mtnip-3* mRNA is not compromised and *mtnip-3* confers chlorate sensitivity in *Atnrt1.1* mutants, demonstrating nitrate transport by this mutant in heterologous assay systems (Bagchi et al., 2012; Salehin et al., 2013). Thus these systems cannot be used to reliably assess potential structural contributions of motif A to MtNPF1.7 function. We first tested the MtNPF1.7_E171K mutant to see if it would reproduce the *mtnip-3* phenotype. The complementation experiment showed that E171K was not able to completely rescue the phenotype, reproducing the *mtnip-3* phenotype previously described (Figures 5A-C and Supplementary Figures 4B, 5G-I, 6B). We then tested the single mutants D93A, R98A, and E171A. All these mutants resulted in completely rescued wild-type phenotype (Figures 5A-C and Supplementary Figures 4B, 5G-I, 6B). This is a surprising result as Asp93 is a strictly conserved residue in NPFs and mutagenesis studies in other NPFs or POTs resulted in inactive transporters. We then proceeded to test double and triple mutants. Mutating Asp93 to alanine in combination with E171A resulted in lack of complementation and a phenotype similar to *mtnip-3* (Figures 5A-C and Supplementary Figures 4B, 5G-I, 6B). A stronger mutated phenotype was observed in the D93R/E171K double mutant (Figures 5D–F and Supplementary Figures 4B, 5J-L, 6C) which is not surprising as in this mutant all the three residues are positively charged. In contrast, R98D/E171K showed complementation. This latter result could be explained by the fact that now E171K can form a salt bridge between TMH3 and TMH4 although with swapped charges with respect to the wild-type transporter (Figures 5D–F and Supplementary Figures 4B, 5J-L, 6C). Finally, the triple mutant D93R/R98D/E171K, designed to rebuild the putative salt bridge(s) with all three individual charges swapped to the opposite charge, was able to restore the wild-type phenotype (Figures 5D–F and Supplementary Figures 4B, 5J-L, 6C) consistent with a putative salt bridge triad that can tolerate a change in the distribution of charges.

### *In silico* Analysis Combined With *in planta* Complementation to Probe the Role of Ala497 in *MtNPF1.7*

The *mtnip-1* mutant, used for our complementation studies, contains a missense mutation, A497V (Yendrek et al., 2010) that results in severe defects in nodules and roots (Veereshlingam et al., 2004). Functional analysis showed that when the mRNA for the *mtnip-1* mutant was injected in oocytes, nitrate was not transported (Bagchi et al., 2012).

**FIGURE 6:**
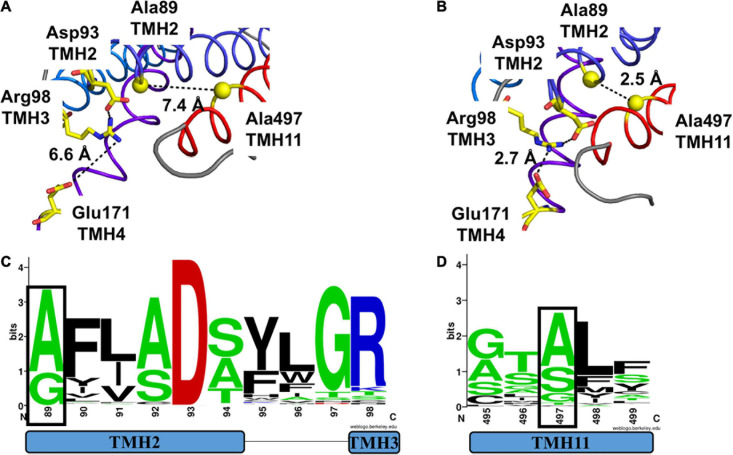
Comparison of packing of TMH2 and TMH11 in MtNPF1.7 models in two different conformations and logo of conservation of the residues needed for packing. Comparison of the conformation of residues belonging to Motif A in the **(A)** I_o_ conformation and **(B)** O_o_ conformation. Ala89 and Ala497 are shown as spheres corresponding to their Ca atoms. Side chains for Asp93, Arg98, and Glu171 are shown as sticks. Broken lines indicate the distance between the closest atoms and possible polar contacts when distance is under 3.6 Å. **(C)** Logo of the conservation of amino acids in Motif A with residue +1 (corresponding to Ala89 in MtNPF1.7) in a black box. **(D)** Logo of the conservation of amino acids in TMH11 with Ala497 in a black box. See Figure 4 for description.

Our analysis of the I_o_ structural model for MtNPF1.7 revealed that Ala497 is located on TMH11 with the closest residues being Pro163 on TMH4 at 3.8Å. However, in the O_o_ structural model conformational changes bring TMH11 closer to TMH2 and Ala497 is packed against Ala89 from TMH2 at 3.2 Å (Figure 6B). The distance between Ala497 and Ala89 in the I_o_ conformation was 7.4 Å (Figure 6A). The distance between Ala497 and Pro163 does not change in the two conformations (not shown).

Interestingly, in the transporter YajR, the template for MtNPF1.7 in the O_o_ conformation, the corresponding residues on TMH2 and TMH11 are two glycines (Gly69 and Gly337) that when mutated to bulky amino acids, resulted in a non-functional protein (Jiang et al., 2013). The YajR crystal structure confirms the formation of the inter-domain helical bundle between TMH2 and TMH11 (Supplementary Figure 2B) that appears to be essential for the stability of the O_o_ conformation. Similarly, in another MFS protein, LacY, two tightly interacting Gly-Gly pairs, this time on the periplasmic side of the transporter, allowed close packing of TMH2 with TMH11 and TMH5 with TMH8 stabilizing the periplasmic-closed conformation (Smirnova et al., 2013; Jiang et al., 2016). These observations support the hypothesis that packing of TMH2 and TMH11 either on the cytoplasmic or the periplasmic side of MFS transporters is essential for the stability of one of the conformations in at least some transporters of the superfamily.

The Gly/Ala residues on TMH2 are part of the conserved Motif A we discussed in the previous section and are located at position 1 of the motif (Figure 6C). Our analysis of plant NPFs sequences shows that only alanines or glycines are allowed on TMH2 at this position. The Gly/Ala residues on TMH11 are less conserved in plant NPFs with serines and threonines also allowed (Figure 6D).

**FIGURE 7:**
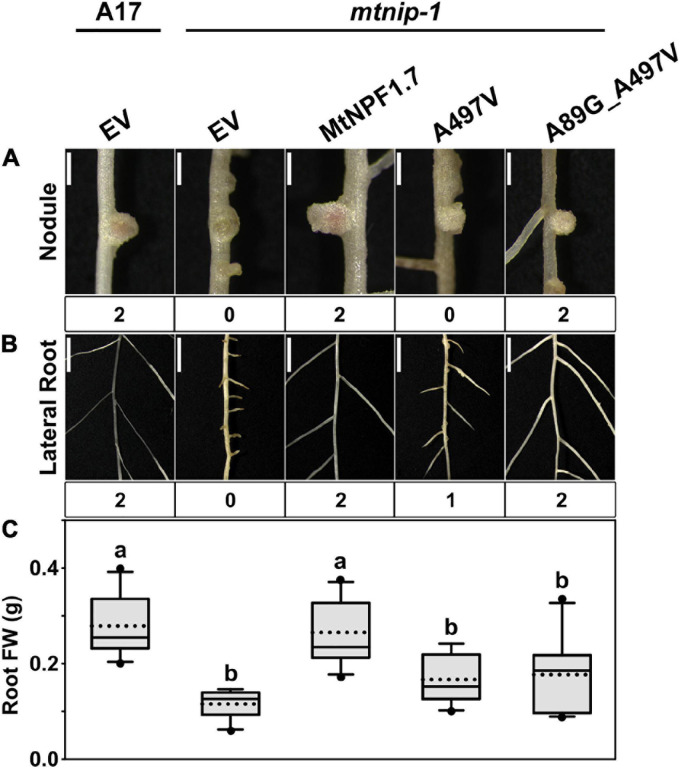
Complementation studies of the *mtnip-1* mutant and restored activity. Images showing the phenotypes in **(A)** nodules and **(B)** lateral roots of (from left to right) A17 plant roots transformed with pMU06 empty vector (EV), *mtnip-1* plant roots transformed with EV, MtNPF1.7_wt (pYCY400), _A497V (pYCY401), and _A89G_A497V (pYCY420). Nodules and lateral roots are evaluated by complementation levels shown below each image as described in Figure 2. **(A)** Bars = 1 mm. **(B)** Bars = 5 mm. **(C)** Plot of root fresh weight from ten independent transgenic plants. See Figure 2D for statistical details.

Since the A497V mutation is known to cause severe defects in roots and nodules (Veereshlingam et al., 2004; Yendrek et al., 2010), we hypothesized that the bulker residue on TMH11 may interfere with the packing of TMH11 with TMH2 in the O_o_ conformation resulting into an inactive transporter. Therefore, we introduced a glycine in position 89 and created the double mutant A89G-A497V with the expectation that a smaller side chain on TMH2 might compensate for the larger size of the valine mutant on TMH11. When we transformed the *mtnip-1* plants with the A89G-A497V double mutant, the wild-type phenotype was rescued with pink nodules and wild type-appearing lateral roots. However, the *mtnip-1* plants with the A89G-A497V double mutant had smaller nodules and lower root mass than wild-type controls (Figure 7 and Supplementary Figures 4B, 5M-O, 6D). Together this suggests that reducing the size of the residue on TMH2 does allow better TMH2-TMH11 packing into the O_o_ conformation, but not quite restoring it fully to wild-type. This likely reflects relative size of the A497V mutation to the A89G mutation.

## Discussion

MtNPF1.7 is required for establishment and persistence of nodule and lateral root meristems as well as primary root meristems in *M. truncatula* (Veereshlingam et al., 2004; Bright et al., 2005; Yendrek et al., 2010). MtNPF1.7 transports nitrate with high affinity: experimentally it has been shown that it confers this activity when expressed in *X. laevis* oocytes and complements the chlorate resistance phenotype when expressed in *A. thaliana nrt1.1* mutants (Bagchi et al., 2012; Salehin et al., 2013). However, it is difficult to reconcile MtNPF1.7’s biological activity solely with nitrate transport because of the following observations. First, nitrate is not essential for growth of legumes, which can use symbiotic nitrogen fixation as their sole N source. Second, the phenotypes found in *mtnpf1.7* mutants: profoundly stunted nodules and lateral roots, and primary roots with aberrant elongation, are only partially rescued by media supplemented with nitrate (Bagchi et al., 2012). Third, *AtNPF6.3*, encoding the well-characterized nitrate transporter, complements *mtnip-1* mutants for their lateral root, but not their nodulation phenotype (Bagchi et al., 2012). However, because AtNPF6.3 also transports auxin (Krouk et al., 2010), this result may not arise from nitrate transport. Fourth, the root architecture phenotypes, which include high concentrations of reactive oxygen species, can be partially rescued by the addition of high concentrations of abscisic acid (Zhang et al., 2014). Fifth, expression of the *MtNPF1.7_E171K* (*mtnip-3*) mutant gene confers nitrate transport in *X. laevis* oocytes and complements the chlorate resistance phenotype in *Atnrt1.1* mutants as well as the wild type *MtNPF1.7* does, even though it has a distinctive nodule and root phenotype (Teillet et al., 2008; Bagchi et al., 2012; Salehin et al., 2013; this work). It has been suggested that MtNPF1.7 could function as a transceptor (Gojon et al., 2011) potentially by co-transporting or alternately transporting a phytohormone (Harris and Dickstein, 2010), which has been shown to occur with other NPF proteins (Chiba et al., 2015; Corratgé-Faillie and Lacombe, 2017; Léran et al., 2020).

In this study, we combined structural modeling of specific residues involved in MtNPF’s function as a transporter with *in planta* experiments to extend our knowledge of the role of these residues in biological function. It was important to assess these residues *in planta* because, as mentioned above, one of the mutants is known to transport nitrate in heterologous assay systems, yet still have defects in biological function. Thus, testing in those systems would risk missing important clues to important residues and structures for function. We obtained structural models of MtNPF1.7 in two different conformations, the O_o_ and I_o_ conformations based on what we know about its biochemical function as a nitrate transporter. These studies, by definition, cannot address unknown biochemical activities in MtNPF1.7 that contribute to biological function, but can help to pinpoint relevant amino acids and potential mechanisms for future assessment. These models helped us identify residues that are potentially involved in the conformational transitions that are at the basis of the alternate access mechanism. We then experimentally tested our hypothesis by using functional complementation of *M. truncatula* plants harboring a mutated *MtNPF1.7*, with plasmids carrying the gene for the wild type or mutated MtNPF1.7 driven by the constitutive *A. thaliana EF1a* promoter. Overall, we observed that the mutations studied here affected nodulation and lateral roots similarly, suggesting that they result from the same function, or lack thereof, in MtNPF1.7 or its mutants.

### *MtNPF1.7* Strictly Requires the First Glutamic Acid Residue From the ExxE(R/K) Motif

It has been observed that MtNPF1.7 requires a proton gradient for active transport of nitrate in experiments performed using oocytes as a heterologous system (Bagchi et al., 2012). Among the candidates for proton transport are proton-titratable residues inside the central cavity belonging to the conserved ExxE(R/K) motif. The role of these residues in NPFs (Ho and Frommer, 2014; Parker and Newstead, 2014; Sun et al., 2014) and in POTs (Solcan et al., 2012; Doki et al., 2013; Zhao et al., 2014; Fowler et al., 2015) has been tested in heterologous systems by site-directed mutagenesis and results support the hypothesis of their involvement in proton transport.

Here, we mutated each of the chargeable residues of the motif and we tested the mutants *in planta* for complementation. Based on previous results on NPFs and POTs, we expected that mutating any of the ExxE(R/K) motif chargeable residues would result in an inactive transporter unable to complement. Instead, our experiments showed that only mutating the first glutamic acid of the motif resulted in non-complementation of the *mtnip-1* mutant, while mutating the second glutamic acid and the lysine of the motif resulted in complementation (Figure 2). These data suggest that, in MtNPF1.7, Glu41 (E_1_) is an essential residue for function, while Glu44 (E_2_) and Lys45 (R/K) are not. The need for at least one glutamic acid in the ExxE(R/K) motif is consistent with the hypothesis that MtNPF1.7 transports nitrate, and potentially a second substrate, through a proton-fueled mechanism *in planta* with E_1_ (E41) being the residue with the essential role. However, since the E_2_ and R/K residues have been shown to be essential for activity in AtNPF6.3 (Ho and Frommer, 2014; Sun et al., 2014) we propose a difference in mechanism between MtNPF1.7 and AtNPF6.3, particularly with respect to the stoichiometry of protons transported per nitrate transport cycle. We suggest the possibility that only one proton is required for transport and that, in MtNPF1.7, nitrate transport could be electrically neutral, with one proton and one negatively charged nitrate transported per transport cycle. This will be difficult to assess experimentally because net charge transfer is required for electrochemical measurements of transport (Tsay et al., 1993; Jeong et al., 2004). This result could also be obtained if the proteins behave differently in plants as compared to heterologous systems. Testing the MtNPF1.7 ExxE(R/K) mutants in a heterologous system such as oocytes may help confirm this hypothesis. Another possibility is that that E41 is strictly needed because it may have an additional role in substrate binding, interrupting not only proton-activated transport but also counterflow when mutated. On the other hand, since mutating E_2_(E44) does not affect the transporter functionality, it is possible that counterflow transport of nitrate in maintained in the MtNPF1.7_E44A mutant and may be sufficient to complement the *mtnip-1* mutation partially or completely, restoring the wild-type phenotype. Relevant to the roles of amino acids in the ExxE(R/K) is the observation that some variants of the ExxE(R/K) motif do exist with a non-fully conserved motif. For instance, the *E. coli* YjdL has a variant QxxEY which can still transport peptides (Aduri et al., 2015). Taken together, this could fit into a new working hypothesis for the ExxE(R/K) motif in MtNPF1.7 where the first glutamic acid has a role both as a proton and a substrate transporter. Alternatively, it is possible that other residues besides E44 in the ExxE(R/K) motif are involved in proton transport. Further experiments are needed to support either hypothesis.

### Residues Supporting the Putative TMH4-TMH10 Salt Bridge Are Not Essential in *MtNPF1.7*

A working model for the alternating-access mechanism in POTs, and consequently in NPFs, includes the formation of a putative salt bridge between oppositely charged amino acids located on TMH4 and TMH10. The salt bridge has been proposed to form in the O_o_ conformation and is considered to be responsible for the stabilization of this conformation. The model is based on mutagenesis studies in NPFs and POTs that showed a lack of proton-driven transport when either one of the two residues was mutated. However, the hypothesis has not been confirmed by crystallography due to the lack of POTs or NPFs structures solved in this conformation. Interestingly, phylogenetic analysis (Longo et al., 2018) previously predicted that residues in these positions are highly conserved in plant NPFs.

We used the structure of a transporter member of the MFS family, YajR, whose structure was solved in the O_o_ conformation as a template to build a model for MtNPF1.7 in this conformation. Surprisingly, we observed that in the MtNPF1.7 model in the O_o_ conformation, the two residues are more distant than in the I_o_ conformation. This observation challenges the proposed working model. Also challenging the model are our mutagenesis results *in planta*. Mutating the charged residues that form the putative TMH4-TMH10 salt bridge to alanines did not disrupt MtNPF1.7’s activity in our assay system. However, swapping the charges of both residues resulted in an inactive protein suggesting that the putative salt bridge cannot be restored with switched charges. These results are surprising and seemingly contradictory. Thus far, our only explanation for this result is that Arg162 and Glu472 have a role in the MtNPF1.7 transport mechanism that is not essential to function, but can be prevented by altering the charge of at least one of their residues. Future experiments will explore mutating these residues one at a time to different charged amino acids.

### Motif A Residues Are Essential to *MtNPF1.7* Function and May Have a Role in Conformational Transition

Although the role of residues from motif A has been studied in several MFS transporters, there were no studies for POTs or NPFs. Our investigation for their role in NPFs was prompted by the realization that both the *mtnip-3* and the *mtnip-1* mutations could interfere with motif A function. Motif A is an MFS-specific motif located at the loop region connecting TMH2 and TMH3 on the cytoplasmic side of the transporters. In the O_o_ conformation, captured in the YajR structure, a charge-relay system was observed between two residues from the motif, Asp73 and Arg77, and a third residue belonging to TMH4, Asp126 (Jiang et al., 2013). The D73R mutation in YajR decreased the melting temperature and increased the occupancy of the I_o_ conformation supporting the hypothesis that the interaction between residues belonging to motif A stabilizes the O_o_ conformation of YajR (Jiang et al., 2013). Mutagenesis studies in a number of other MFS proteins have shown that conservation of residues from Motif A is required for the proper function of the proteins (Supplementary Table 5 and references therein). Our analysis of sequences from plant NPFs shows that Motif A residues corresponding to the charge-relay system are highly conserved in NPFs indicating that also in NPFs these residues may have an important role in stabilizing the O_o_ conformation. In MtNPF1.7, these residues correspond to Asp93 and Arg98, predicted to interact with Glu171. The latter residue, outside of Motif A, corresponds to the residue mutated in *mtnip-3*. When we mutated Motif A residues in MtNPF1.7, we observed that mutating only one of the two residues to alanine restored functionality *in planta*, but the double mutants with the mutated Glu171 residue on TMH4 resulted in a non-functional transporter. Some of these results are unexpected as Asp93 is strictly conserved and several studies have shown that mutating this residue results in inactive proteins. It may be that the difference observed in mutagenized proteins reflect the differences between mutating the residue to one with no charge as distinct from changing the charge. One hypothesis is that *in planta* the protein may have residual activity when only one of the residues is mutated, and that heterologous systems are differently sensitive to mutagenesis of these residues.

Another residue from Motif A at position +1 (Ala89 in MtNPF1.7) has been shown to have a role in the stability of the O_o_ conformation allowing tight helix-helix packing between TMH2 and TMH11 in this conformation. In our studies we started with a mutation on TMH11 (A497V) that causes the defective *mtnip-1* phenotype in *Medicago*. Mutating Ala89 to glycine restored the protein’s activity indicating that a smaller residue on TMH2 can compensate for the presence of a bulkier amino acid residue on TMH11. In our work, we were guided by previous studies on YajR where only residues with small side chains are tolerated in motif A position +1, with the introduction of a bulkier side chain resulting into a disturbed O_o_ conformation in YajR and inactive transport (Jiang et al., 2013). Studies in other MFS transporters support this model indicating that motif A regulates conformational changes in MFS proteins by balancing the stability between O_o_ and I_o_ conformations.

Although the role of Motif A residues had been studied in several MFS transporters, there are no studies for POTs or NPFs. This is the first study that investigates their role in NPFs and support a role of motif A function as a molecular switch that regulates conformational changes in MtNPF1.7 and likely in other NPFs.

### Proton-Driven vs. Counterflow

Functional studies of NPFs and POTs in heterologous systems have shown that counterflow transport can be maintained even when residues essential to proton-driven transport are mutated. Although counterflow transport is not as efficient as proton-driven transport and results in a reduced transport capability, these results suggest that ligand-driven transport is still possible in the absence of a proton-driven force. However, some residues are strictly required for both proton-driven transport and counterflow. For example, in PepT_*St*_ when Tyr30, Glu300, or Glu400 are mutated to alanines, the transporter does not uptake di-alanine in either proton-driven or peptide-driven counterflow transport (Solcan et al., 2012). This suggests that transporters maintain their ability to undergo the conformational changes needed by the transporter to alternatively open outwards or inwards. In such cases the alternating-access mechanism is driven by the substrate with transport direction depending on the concentration of the substrate on the two sides of the membrane. Transporters become uniporters instead of symporters.

Interestingly, when conserved residues of Motif A are mutated, both proton-driven and counterflow activity are abolished. This may happen because the protein is essentially frozen in the I_o_ conformation and it is not able to open towards the extracellular space. Essentially, the alternating-access mechanism is deeply compromised.

### A Model for *MtNPF1.7* Alternating-Access Mechanism

An alternating-access mechanism has been proposed for MFS transporters including NPFs and POTs in which the substrate and the proton(s) binding sites are alternatively accessible from either the outside, with the transporter in the O_o_ conformation, or the inside, in the I_o_ conformation, of the membrane (Yan, 2015; Zhang et al., 2015; Quistgaard et al., 2016). Such a mechanism has been described from an energetic point of view with the O_o_ conformation seen as the ground state and the I_o_ conformation as the excited state. The transition from the ground state to the excited state is driven by protonation inside the central cavity, whereas the transition from excited to ground is more or less automatic. The two conformations are stabilized by salt bridges that form between TMHs either intra- or inter-domains.

Some structural elements are conserved among MFS proteins and provide a common transition mechanism while other elements are only present in some subfamilies indicating that each may have evolved specific mechanisms. For instance, proton-binding residues are distributed differently in the access cavity of MFS transporters belonging to different families. So while in NPFs and POTs protons bind to residues located in the ExxE(R/K) motif, such motif is not found in other MFS transporters. Therefore, transitions between conformations are guided by specific amino acids that may differ between different transporters within the superfamily. Which specific residues are involved and the details of the conformational changes involved are still subject to investigation.

In the case of MtNPF1.7, results presented in this work suggest a fundamental role for residues from motif A in stabilizing the O_o_ conformation and a relevant role for the first glutamic acid in the ExxE(R/K) motif.

## Summary and Future Prospects

In this work, we investigated the role of selected residues from MtNPF1.7 in the general mechanisms of proton-driven MFS symporters, including substrate-binding coupled to protonation, regulation of motif A-based switches, and salt bridges. Our results *in planta* may indicate that counterflow transport is insufficient to restore wildtype phenotype by complementation, but could restore MtNPF1.7 transport in heterologous systems consistent with previous results in oocytes. The results underline the potential of motif A to contribute to NPF transport mechanisms generally. Further studies, especially those that focus on specific transport assays in heterologous systems, are required to support our hypothesis and answer outstanding questions regarding the role of MtNPF1.7 in nitrate transport and nodulation.

## Data Availability Statement

The original contributions presented in the study are included in the article/Supplementary Material, further inquiries can be directed to the corresponding author.

## Author Contributions

AL conducted the structural modeling and analysis. Y-CY conducted the cloning and *in planta* experiments. Y-CY, RD, and AL interpreted the data and wrote the manuscript. All authors designed the experiments, contributed to the article, and approved the submitted version.

## Conflict of Interest

The authors declare that the research was conducted in the absence of any commercial or financial relationships that could be construed as a potential conflict of interest.

## Footnotes

1 http://weblogo.berkeley.edu/

2 https://swissmodel.expasy.org/

3 https://opm.phar.umich.edu/
