# Supplementary material for: Structural Modeling and in planta Complementation Studies Link Mutated Residues of the Medicago truncatula Nitrate Transporter NPF1.7 to Functionality in Root Nodules
Source: Front Plant Sci. 2021 Jul 1;12:685334. doi: 10.3389/fpls.2021.685334 (PMC8282211; doi:10.3389/fpls.2021.685334)
Supplement: Supplementary file 1 [file Data_Sheet_1.pdf]

Supplementary Data

Front. Plant Sci. | doi: 10.3389/fpls.2021.685334

Structural modeling and *in planta* complementation studies link mutated residues of the *Medicago truncatula* nitrate transporter NPF1.7 to functionality in root nodules.

Yao-Chuan Yu, Rebecca Dickstein[†], Antonella Longo[†]*

Department of Biological Sciences and BioDiscovery Institute, University of North Texas, Denton, TX, United States

[†]These authors have contributed equally to this work and share senior authorship.

*** Correspondence:**

Corresponding Author

antonella.longo@unt.edu

Keywords:

Medicago truncatula, NPF transporters, NIP/LATD, symbiotic nitrogen fixation, root architecture, nitrate signaling

Supplementary Table S1. List of available crystal structures for plant NPFs, bacterial POTs, and relevant MFSs. Abbreviations: cm, chloramphenicol; DXC, deoxycholate; LDAO, n-dodecyl-N,N-dimethylamine-N-oxide

Protein	Organism	Conformation	Ligand	PDB ID	Reference
**AtNPF6.3**	*Arabidopsis thaliana*	Inward-open<br>Inward-open<br>Inward-open	None<br>Nitrate<br>Nitrate	5A2N<br>5A2O<br>4OH3	Parker & Newstead, 2014<br><br>Sun et al., 2014
**PepT$_{So}$**	*Shewanella oneidensis*	Occluded state<br>Inward-open	None<br>None	2XUT<br>4UVM	Newstead et al., 2011<br>Fowler et al., 2015
**PepT$_{So2}$**	*Shewanella oneidensis*	Inward-open<br>Inward-open<br>Inward-open<br>Inward-open	Alafosfalin<br>Ala-Ala-Ala<br>Ala-Tyr-(Br)<br>Ala-Tyr-(Br)-Ala	4LEP<br>4TPJ<br>4TPH<br>4TPG	Guettou et al., 2013<br>Guettou et al., 2014
**PepT$_{St}$**	*Streptoccus thermophilus*	Inward-open<br>Inward-open<br>Inward-open<br>Inward-open<br>Inward-open<br>Inward-open<br>Inward-open<br>Inward-open<br>Inward-open<br>Inward-open<br>Inward-open<br>Inward-open<br>Inward-open<br>Inward-open<br>Inward-open<br>Inward-open<br>Inward-open<br>Inward-open	None<br>None<br>Ala-Phe<br>Ala-Ala-Ala<br>None<br>None<br>Ala-Phe<br>Ala-Phe<br>None<br>Ala- Gln<br>Ala- Leu<br>Asp-Glu<br>Phe-Ala<br>HEPES<br>Phosphate<br>none<br>HEPES<br>Phe-Ala-Gln	4APS<br>4D2B<br>4D2C<br>4D2D<br>4XNI<br>4XNJ<br>5D58<br>5D59<br>5MMT<br>5OXK<br>5OXL<br>5OXM<br>5OXN<br>5OXQ<br>5OXP<br>5OXO<br>6EIA<br>6GHJ	Solcan et al., 2012<br>Lyons et al., 2014<br><br><br>Huang et al., 2015<br><br>Huang et al., 2016<br><br>Quistgaard et al., 2017<br>Martinez Molledo et al., 2018a<br><br><br><br><br><br><br><br>Martinez Molledo et al., 2018b
**GkPOT**	*Geobacillus kaustophilus*	Inward-open<br>Inward-open	None<br>Alafosfalin	4IKV<br>4IKZ	Doki et al., 2013
**YbgH**	*Escherichia coli*	Inward-open	None	4Q65	Zhao et al., 2014
**YePEPT**	*Yersinia enterocolitica*	Inward-open	None	4W6V	Boggavarapu et al., 2015
**PepT$_{Xc}$**	*Xanthomonas campestris*	Auto-inhibited	None	6EI3	Parker et al., 2017
**YajR**	*Escherichia coli*	Outward-open	none	3WDO	Jiang et al., 2013
**MdfA**	*Escherichia coli*	Inward-open<br>Inward-open<br>Inward-open<br>Outward-open	Cm<br>DXC<br>LDAO<br>none	4ZOW<br>4ZP0<br>4ZP2<br>6GV1	Heng et al., 2015<br><br><br>Nagarathinam et al., 2017

Supplementary Table S2. List of plasmids used for this study.
Abbreviations: Amp^R, ampicillin resistance; Kan^R, kanamycin resistance; Sm^R, streptomycin resistance; Sp^R, spectinomycin resistance.

Plasmid	Description	Purpose	Backbone	Selection	Reference
**pcDNA3.1-nipc**	pCMV-MtNPF1.7	MtNPF1.7 cDNA cloning	pcDNA3.1(-)	Amp^R	Bagchi et al, 2012
**pMS210**	pEF1α-MtNPF1.7-7Ala-eGFP	pEF1α-MtNPF1.7-eGFP cloning	pUC19	Amp^R	Bagchi et al, 2012
**pMS217**	pEF1α-Mtnip-1-7Ala-eGFP	Mtnip-1 cDNA cloning	pCambia 2301	Kan^R	Dickstein's collection
**pMS219**	pEF1α-Mtnip-3-7Ala-eGFP	Mtnip-3 cDNA cloning	pCambia 2301	Kan^R	Dickstein's collection
**pYCY200**	MtNPF1.7_WT	Site-directed mutagenesis	pGEMTeasy	Amp^R	This study
**pYCY201**	Mtnip-1 (MtNPF1.7_A497V)	Site-directed mutagenesis	pGEMTeasy	Amp^R	This study
**pYCY202**	Mtnip-3 (MtNPF1.7_E171K)	Site-directed mutagenesis	pGEMTeasy	Amp^R	This study
**pYCY203**	MtNPF1.7_E41A	Site-directed mutagenesis	pGEMTeasy	Amp^R	This study
**pYCY204**	MtNPF1.7_E44A	Site-directed mutagenesis	pGEMTeasy	Amp^R	This study
**pYCY205**	MtNPF1.7_K45A	Site-directed mutagenesis	pGEMTeasy	Amp^R	This study
**pYCY206**	MtNPF1.7_K45R	Site-directed mutagenesis	pGEMTeasy	Amp^R	This study
**pYCY207**	MtNPF1.7_R162A	Site-directed mutagenesis	pGEMTeasy	Amp^R	This study
**pYCY208**	MtNPF1.7_E472A	Site-directed mutagenesis	pGEMTeasy	Amp^R	This study
**pYCY209**	MtNPF1.7_R162E	Site-directed mutagenesis	pGEMTeasy	Amp^R	This study
**pYCY210**	MtNPF1.7_E472R	Site-directed mutagenesis	pGEMTeasy	Amp^R	This study
**pYCY211**	MtNPF1.7_R162EE472R	Site-directed mutagenesis	pGEMTeasy	Amp^R	This study
**pYCY212**	MtNPF1.7_D93A	Site-directed mutagenesis	pGEMTeasy	Amp^R	This study
**pYCY213**	MtNPF1.7_R98A	Site-directed mutagenesis	pGEMTeasy	Amp^R	This study
**pYCY214**	MtNPF1.7_E171A	Site-directed mutagenesis	pGEMTeasy	Amp^R	This study
**pYCY215**	MtNPF1.7_D93AE171A	Site-directed mutagenesis	pGEMTeasy	Amp^R	This study
**pYCY216**	MtNPF1.7_R98AE171A	Site-directed mutagenesis	pGEMTeasy	Amp^R	This study
**pYCY217**	MtNPF1.7_D93RE171K	Site-directed mutagenesis	pGEMTeasy	Amp^R	This study
**pYCY218**	MtNPF1.7_R98DE171K	Site-directed mutagenesis	pGEMTeasy	Amp^R	This study
**pYCY219**	MtNPF1.7_D93RR98DE171K	Site-directed mutagenesis	pGEMTeasy	Amp^R	This study
**pYCY220**	MtNPF1.7_A89GA497V	Site-directed mutagenesis	pGEMTeasy	Amp^R	This study
**pYCY300**	pEF1α-MtNPF1.7_WT-eGFP	Expression construct assembly	pGEMTeasy	Amp^R	This study
**pYCY301**	pEF1α-MtNPF1.7_A497V-eGFP	Expression construct assembly	pGEMTeasy	Amp^R	This study
**pYCY302**	pEF1α-MtNPF1.7_E171K-eGFP	Expression construct assembly	pGEMTeasy	Amp^R	This study

**pYCY303**	pEF1α-MtNPF1.7_E41A-eGFP	Expression construct assembly	pGEMTeasy	Amp^R	This study
**pYCY304**	pEF1α-MtNPF1.7_E44A-eGFP	Expression construct assembly	pGEMTeasy	Amp^R	This study
**pYCY305**	pEF1α-MtNPF1.7_K45A-eGFP	Expression construct assembly	pGEMTeasy	Amp^R	This study
**pYCY306**	pEF1α-MtNPF1.7_K45R-eGFP	Expression construct assembly	pGEMTeasy	Amp^R	This study
**pYCY307**	pEF1α-MtNPF1.7_R162A-eGFP	Expression construct assembly	pGEMTeasy	Amp^R	This study
**pYCY308**	pEF1α-MtNPF1.7_E472A-eGFP	Expression construct assembly	pGEMTeasy	Amp^R	This study
**pYCY309**	pEF1α-MtNPF1.7_R162E-eGFP	Expression construct assembly	pGEMTeasy	Amp^R	This study
**pYCY310**	pEF1α-MtNPF1.7_E472R-eGFP	Expression construct assembly	pGEMTeasy	Amp^R	This study
**pYCY311**	pEF1α-MtNPF1.7_R162EE472R-eGFP	Expression construct assembly	pGEMTeasy	Amp^R	This study
**pYCY312**	pEF1α-MtNPF1.7_D93A-eGFP	Expression construct assembly	pGEMTeasy	Amp^R	This study
**pYCY313**	pEF1α-MtNPF1.7_R98A-eGFP	Expression construct assembly	pGEMTeasy	Amp^R	This study
**pYCY314**	pEF1α-MtNPF1.7_E171A-eGFP	Expression construct assembly	pGEMTeasy	Amp^R	This study
**pYCY315**	pEF1α-MtNPF1.7_D93AE171A-eGFP	Expression construct assembly	pGEMTeasy	Amp^R	This study
**pYCY316**	pEF1α-MtNPF1.7_R98AE171A-eGFP	Expression construct assembly	pGEMTeasy	Amp^R	This study
**pYCY317**	pEF1α-MtNPF1.7_D93RE171K-eGFP	Expression construct assembly	pGEMTeasy	Amp^R	This study
**pYCY318**	pEF1α-MtNPF1.7_R98DE171K-eGFP	Expression construct assembly	pGEMTeasy	Amp^R	This study
**pYCY319**	pEF1α-MtNPF1.7_D93RR98DE171K-eGFP	Expression construct assembly	pGEMTeasy	Amp^R	This study
**pYCY320**	pEF1α-MtNPF1.7_A89GA497V-eGFP	Expression construct assembly	pGEMTeasy	Amp^R	This study
**pMU06**	Empty vector	Hairy root transformation	pMU06	Sm^R, Sp^R	Dr. Wei Liu
**pYCY400**	pEF1α-MtNPF1.7_WT-eGFP	Hairy root transformation	pMU06	Sm^R, Sp^R	This study
**pYCY401**	pEF1α-MtNPF1.7_A497V-eGFP	Hairy root transformation	pMU06	Sm^R, Sp^R	This study
**pYCY402**	pEF1α-MtNPF1.7_E171K-eGFP	Hairy root transformation	pMU06	Sm^R, Sp^R	This study
**pYCY403**	pEF1α-MtNPF1.7_E41A-eGFP	Hairy root transformation	pMU06	Sm^R, Sp^R	This study
**pYCY404**	pEF1α-MtNPF1.7_E44A-eGFP	Hairy root transformation	pMU06	Sm^R, Sp^R	This study
**pYCY405**	pEF1α-MtNPF1.7_K45A-eGFP	Hairy root transformation	pMU06	Sm^R, Sp^R	This study
**pYCY406**	pEF1α-MtNPF1.7_K45R-eGFP	Hairy root transformation	pMU06	Sm^R, Sp^R	This study
**pYCY407**	pEF1α-MtNPF1.7_R162A-eGFP	Hairy root transformation	pMU06	Sm^R, Sp^R	This study
**pYCY408**	pEF1α-MtNPF1.7_E472A-eGFP	Hairy root transformation	pMU06	Sm^R, Sp^R	This study
**pYCY409**	pEF1α-MtNPF1.7_R162E-eGFP	Hairy root transformation	pMU06	Sm^R, Sp^R	This study
**pYCY410**	pEF1α-MtNPF1.7_E472R-eGFP	Hairy root transformation	pMU06	Sm^R, Sp^R	This study
**pYCY411**	pEF1α-MtNPF1.7_R162EE472R-eGFP	Hairy root transformation	pMU06	Sm^R, Sp^R	This study
**pYCY412**	pEF1α-MtNPF1.7_D93A-eGFP	Hairy root transformation	pMU06	Sm^R, Sp^R	This study
**pYCY413**	pEF1α-MtNPF1.7_R98A-eGFP	Hairy root transformation	pMU06	Sm^R, Sp^R	This study

**pYCY414**	pEF1α-MtNPF1.7_E171A-eGFP	Hairy root transformation	pMU06	Sm^R, Sp^R	This study
**pYCY415**	pEF1α-MtNPF1.7_D93AE171A-eGFP	Hairy root transformation	pMU06	Sm^R, Sp^R	This study
**pYCY416**	pEF1α-MtNPF1.7_R98AE171A-eGFP	Hairy root transformation	pMU06	Sm^R, Sp^R	This study
**pYCY417**	pEF1α-MtNPF1.7_D93RE171K-eGFP	Hairy root transformation	pMU06	Sm^R, Sp^R	This study
**pYCY418**	pEF1α-MtNPF1.7_R98DE171K-eGFP	Hairy root transformation	pMU06	Sm^R, Sp^R	This study
**pYCY419**	pEF1α-MtNPF1.7_D93RR98DE171K-eGFP	Hairy root transformation	pMU06	Sm^R, Sp^R	This study
**pYCY420**	pEF1α-MtNPF1.7_A89GA497V-eGFP	Hairy root transformation	pMU06	Sm^R, Sp^R	This study

Supplementary Table S3. Primers used for site-directed mutagenesis.
Mutated codons in bold, mutated bases in capitol letters.

**Mutation**	**Primer sequence**
Glu41Ala	NIP_E41A_F atcatagtgaat**gCg**tgtcttgagaaa<br>NIP_E41A_R aaagggcatggttcttaaaccacc
Glu44Ala	NIP_E44A_F aatgagtgtctt**gCg**aaagtggcaagt<br>NIP_E44A_R cactatgataaagggcatggttct
Lys45Ala	NIP_K45A_F gagtgtcttgag**GCa**gtggcaagttat<br>NIP_K45A_R attcactatgataaagggcatggt
Lys45Arg	NIP_K45R_F tgtcttgag**aGa**gtggcaagttatg<br>NIP_K45R_R ctcattcactatgataaaggg
Arg162Ala	NIP_R162A_F tggttgtgtt**GCa**ccttgttcc<br>NIP_R162A_R gctccaattgaaattaatcc
Arg162Glu	NIP_R162E_F tggttgtgtt**GAa**ccttgttcc<br>NIP_R162E_R gctccaattgaaattaatcc
Glu472Ala	NIP_E472A_F ggattcgcc**gCa**gctttcaca<br>NIP_E472A_R aaacaaaataaactctggagcaag
Glu472Arg	NIP_E472R_F tggattcgcc**AGa**gctttcacac<br>NIP_E472R_R aacaaaataaactctggagc
Glu171Lys (nip-3)	NIP_E171K_F gcctttggagca**Aag**caattgactatt<br>NIP_E171K_R tatggaacaaggtctaacacaacc
Glu171Ala	NIP_E171A_F gcctttggagca**gCg**caattgactatt<br>NIP_E171A_R tatggaacaaggtctaacacaacc
Asp93Ala	NIP_D93A_F gcttttctctct**gCC**tcttacttgggt<br>NIP_D93A_R accaaagatggacaaaacattgga
Arg98Ala	NIP_R98A_F tcttacttgggt**GCc**ttcaatgtcatc<br>NIP_R98A_R atcagagagaaaagcaccaaagat
Asp93Arg,Glu171Lys	nip-3_D93R_F ttttctctct**AGA**tcttacttgggtcg<br>nip-3_D93R_R gcaccaaagatggacaaaac
Arg98Asp,Glu171Lys	nip-3_R98D_F ttacttgggt**GAT**ttcaatgtcatcac<br>nip-3_R98D_R gaatcagagagaaaagcac
Asp93Arg,Arg98Asp,Glu171Lys	nip-3_D93R-R98D_F ttacttgggt**GAT**ttcaatgtcatcac<br>nip-3_D93R-R98D_R gatctagagagaaaagcac
Ala89Gly,Ala497Val	nip-1_A89G_F atctttggt**gGt**tttctctctg<br>nip-1_A89G_R ggacaaaacattggacatag

Supplementary Table S4. Other primers used in this study.

**Primer name**	**Sequence**	**Purpose**
11_NcoI_NIP_F	ctgtaacgctaccatggagtacacaaacagtgatg	Cloning
6_NheI_NIP_R	tatcacgctagctgaagtaggcaactccctgtaac	Cloning
6_SalI_NIP_R	tatcacgtcgactgaagtaggcaactccctgtaac	Cloning
pGEMT_Gib_F	gtaggtaccatatgggagagctcccaac	pYCY300 assembly
pGEMT_Gib_R	ctcgagtcgtattacaattcactggc	pYCY300 assembly
pGEMT_EF1α_Gib_F	gtgaattgtaatacgactcgagctctagagaagagtgtcat	pYCY300 assembly
pGEMT_GFP_Gib_R	ttgggagctctcccatatggtacctacttgtacagctcgtc	pYCY300 assembly
eGFP_F	atggtgagcaagggcgagga	Sequencing
NIP_1177_F	accgtgtaggagtacctctactag	Sequencing
pEF1α_703_R	actggatttgttatgctactccag	Sequencing
NIP_826_F	tctgatcgttactgtcaaggtc	Sequencing
NIP_955_R	gatttgaaattgacccatctcg	Sequencing
pEF1α_2F	atcggtttgactccgatagcgg	Sequencing
eGFP_1R	tttacgtcgccgtccagctc	Sequencing

Supplementary Table S5. List of mutagenesis studies of plant NPFs, bacterial POTs, and other transporters belonging to the MFS superfamily.

Transporter	Mutation	System	Reference
**Plant NPFs**			
AtNPF6.3	E41A, E44A, R45A, L49A, K164A, K164D, R264A, R266A, K267A, Q358A, Y388A, E476A, E476D	Oocytes	Ho & Frommer, 2014
AtNPF6.3	E41A, E44A, R45A, K164A, H356A	Oocytes	Sun et al., 2014
AtNPF6.3	H356A	Liposome-based	Parker & Newstead, 2014
AtNPF2.11	E57A, E60A, K61A, T58A, T58R, T58D, F58L, F59R, F59D	Oocytes	Jørgensen et al., 2015
**Bacterial POTs**			
GkPOT	E32Q, R36A, Y40A, R43Q, K136A, N166A, E310Q	Liposome-based	Doki et al., 2013
$PepT_{St}$	E22A, E22Q, E25A, E25Q, R26K, Y29A, Y30A, Y30F, K126A, E400D	Proteoliposome based	Solcan et al., 2012
$PepT_{So}$	E21A, E24A, R25A, D79A, K84A, D136A, K127A, D316A	Liposome-based	Fowler et al., 2015<br>Parker et al., 2017
YjdL	Q17E, Y21R, E20Q, Q17E-Y21R, Q17E-E20Q, E20Q-Y21R, Q17E-E20Q-Y21R	bacteria	Aduri et al., 2015
YbgH	Q18E, E21Q, E21A, Y22A, Y22F, D70N, D70A, E163A, E163Q, R297A, E391Q, D395N, Q18E/Y22R, Q18E/Y22R/D395S, D70A/E163A, E163A/R297A, D70A/R297A/E163A,	bacteria	Zhao et al., 2014
**Bacterial MFSs**			
YajR	G51W, G69W, D73R, R77E, D126R	*in vitro*	Jiang et al., 2013
TetA(P)	D67K, D67E, D67N	bacteria	Kennan et al., 1999

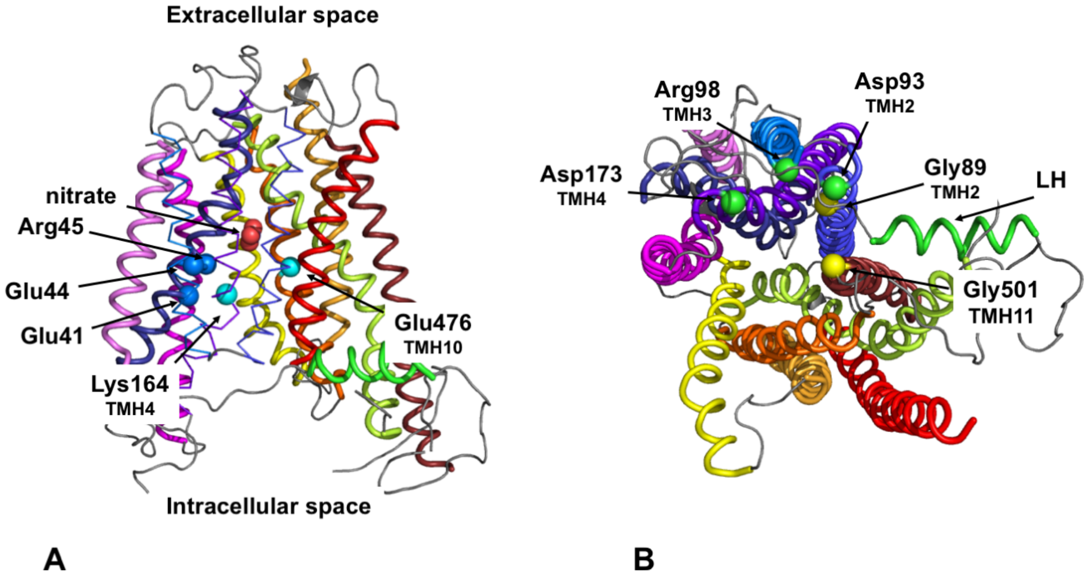

Supplementary Figure S1. Crystal structure of AtNPF6.3 in the I_o conformation.
Loop rendering of the peptide backbone of the crystal structure of AtNPF6.3 (pdb: 4oh3) (Sun et al., 2014). Spheres correspond to Cα atoms. **(A)** The transporter as viewed in the plane of the membrane with the ExxE(R/K) motif residues, Glu41, Glu44, and Arg45, as dark blue spheres, and with residues predicted to form a TMH4-TMH10 salt bridge in the O_o conformation, Lys164 and Glu476, as cyan spheres. The substrate nitrate localized at the bottom of a channel is shown as red spheres. **(B)** Rotation of 90° of **(A)** for a view from the cytoplasmic side of the membrane with residues involved in the salt bridge triad, Asp93, Arg98, and Asp173, as green spheres, and two glycine residues, Gly89 and Gly501, that we hypothesize to be important for TMH2-TMH11 packing in the O_o conformation, as yellow spheres. LH: lateral helix.

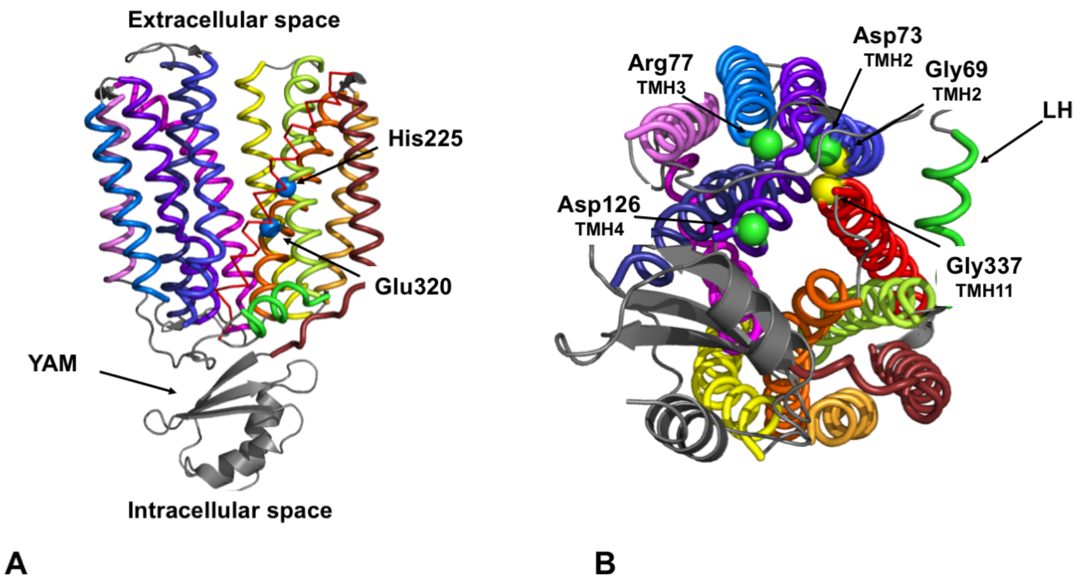

Supplementary Figure S2. Crystal structure of *E. coli* YajR in the O_0 conformation.
Loop rendering of the peptide backbone of the crystal structure of *E. coli* YajR (pdb: 3wdo) (Jiang et al., 2013). Spheres correspond to Cα atoms. **(A)** The transporters as viewed in the plane of the membrane with the 12 TMHs of the MFS core in a gradient of colors and the YAM domain in grey. His225 and Glu320 (blue spheres) have been indicated as potential proton transporters. **(B)** Rotation of 90° of **(A)** for a view from the cytoplasmic side of the membrane. Asp73 and Arg77 (green spheres) belong to Motif A and are part of a salt-bridge triad with Asp126 (green sphere) that stabilizes this conformation. Gly69 and Gly337 (yellow spheres) are important for helix-helix packing in this conformation. LH: lateral helix.

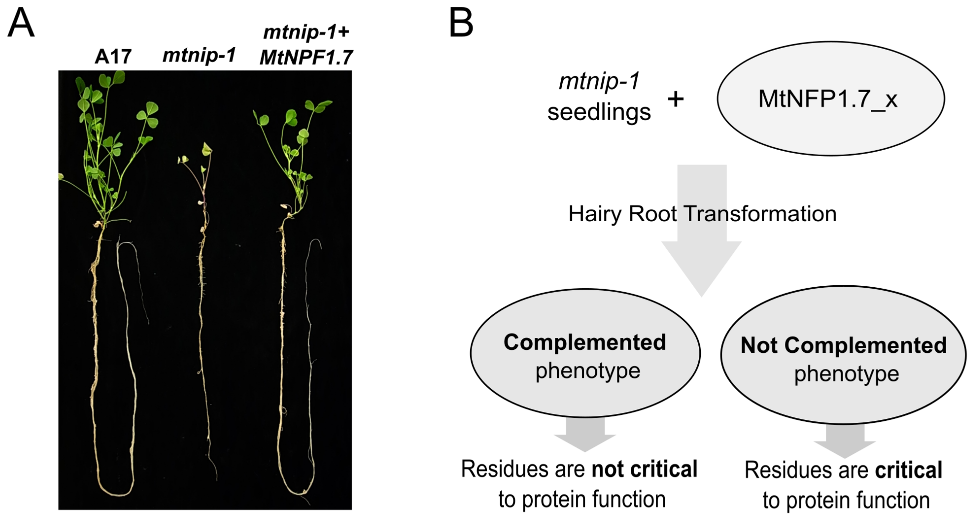

Supplementary Figure S3. Complementation study rationale.
(A) *Medicago truncatula* genotype A17 shows well-developed roots and shoots. The *mtnip-1* shows defective roots and shoots phenotype as well as altered nodulation (not shown). Expressing MtNPF1.7 in *mtnip-1* roots restores the WT-like phenotype and thus complements the defect. **(B)** To screen residues important for MtNPF1.7 function, the MtNPF1.7 residue-substituted variants (MtNPF1.7_x) were expressed in *mtnip-1* plant roots via hairy root transformation. The levels of complementation were evaluated by observing the phenotypes of transformed plants. If the WT-like phenotype is restored (complemented) by the MtNPF1.7_mutant, it indicates that the substituted residue is not critical to protein function. If the phenotype is not complemented, it indicates that the residue is potentially critical to protein function.

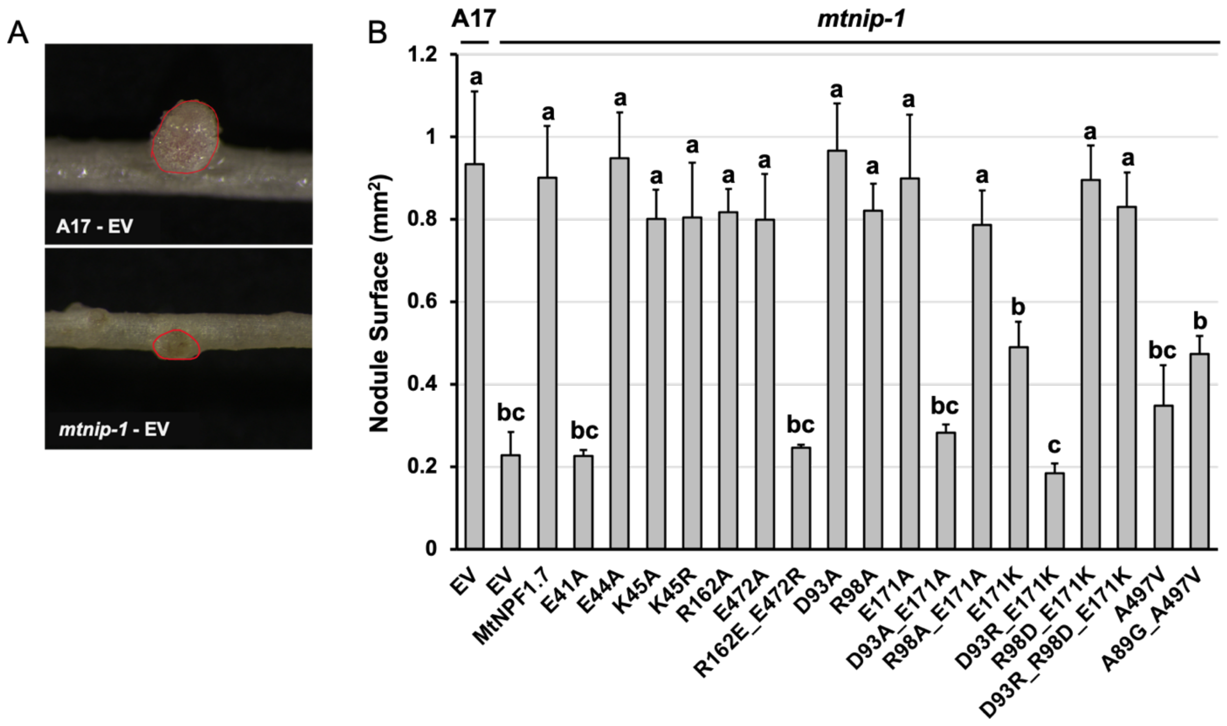

Supplementary Figure S4. Nodule surface area of *in planta* complementation mutants.
(A) Nodule surface was estimated by measuring the nodule/ nodule primordium area (circled in red) in the transformed roots. Examples shown here are A17 (top) and *mtnip-1* (bottom) plant roots transformed with empty vector (EV). **(B)** The nodule surface (mm^2) of A17 or *mtnip-1* plants transformed with EV, *MtNPF1.7*, or amino acid substituted mutants at 15 dpi. The data were collected from three plants per line (n=3) and analyzed with Fiji software (Schindelin et al., 2012). The letters above each bar indicate the significant differences analyzed by ANOVA and the post-hoc Tukey's HSD (honestly significant difference) test ($P < 0.05$). The nodule surface area correlates well with the visual assessment data reported in Figures 2, 3, 5, and 7 except in the case of mutant A89G_A497V. For that case, the visual assessment was scored a "2" because of the presence of pink nodule pigments, indicating that nodules are Fix^+, while the surface area data would categorize mutant A89G_A497V as a "1."

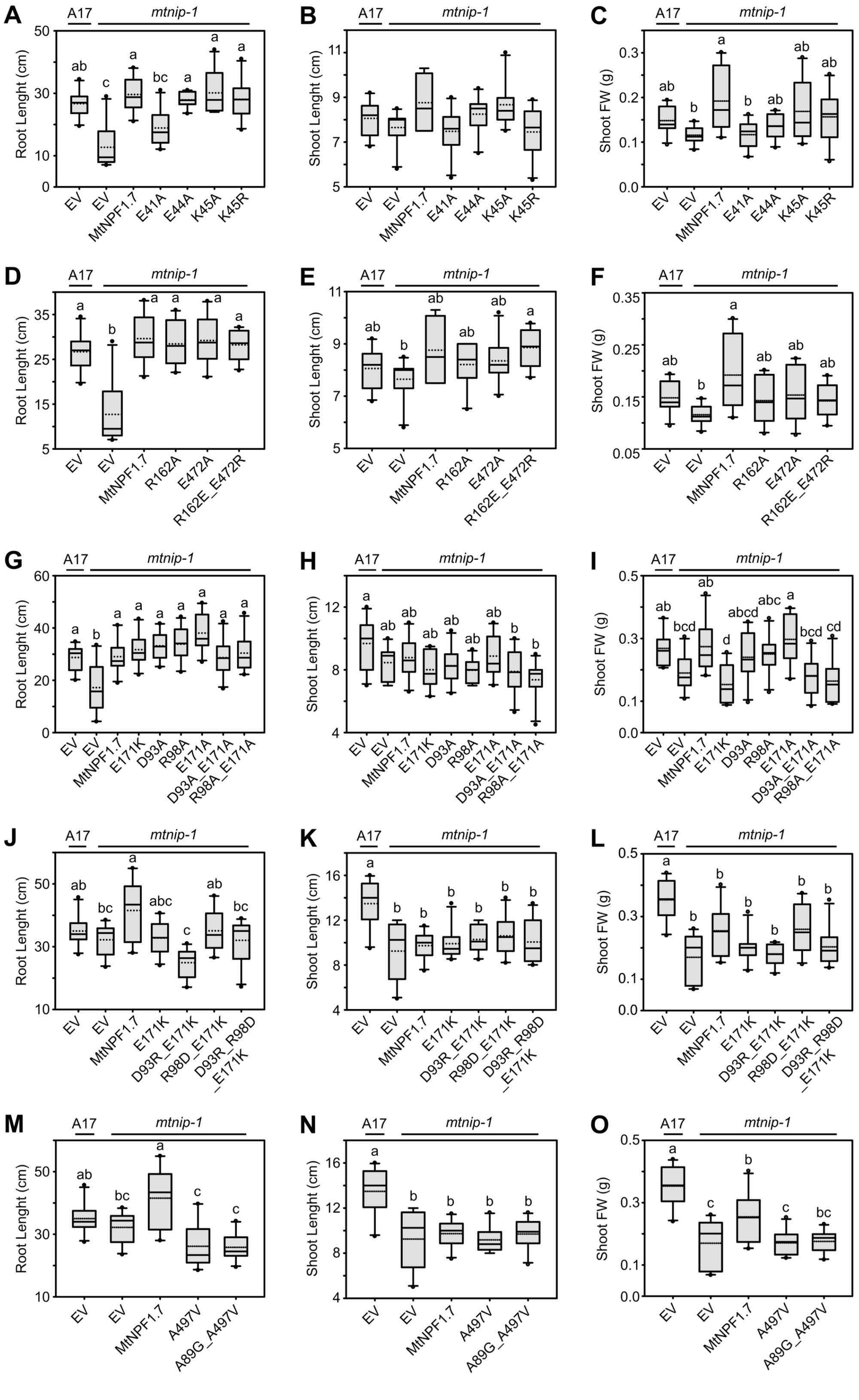
A
B
C
D
E
F
G
H
I
J
K
L
M
N
O
A17
mtnip-1
Root Lenght (cm)
Shoot Lenght (cm)
Shoot FW (g)
EV
MtNPF1.7
E41A
E44A
K45A
K45R
R162A
E472A
R162E_E472R
E171K
D93A
R98A
E171A
D93A_E171A
R98A_E171A
D93R_E171K
R98D_E171K
D93R_R98D _E171K
A497V
A89G_A497V

Supplementary Figure S5. Additional phenotypic data in complementation assay.
Plots of root length **(A, D, G, J, and M)**, shoot length **(B, E, H, K, and N)**, and shoot fresh weight **(C, F, I, L, and O)** of plants expressing empty vector, MtNPF1.7, or MtNPF1.7_mutant variants. Data are shown as box plots (n=10 independent transgenic plants). The lower and upper boundaries of the box indicate the 25th and 75th percentiles, respectively, with the median and mean marked as solid and dotted lines, respectively. The ends of lower and upper whiskers indicate the minimum and maximum values. Outliers are shown as dots. The letters above each box indicate the significant differences analyzed by ANOVA and the post-hoc Tukey's HSD (honestly significant difference) test ($P < 0.05$). Note that plants grouped together were grown together in the same aeroponics chamber with their respective positive and negative control plants to assure validity of data.

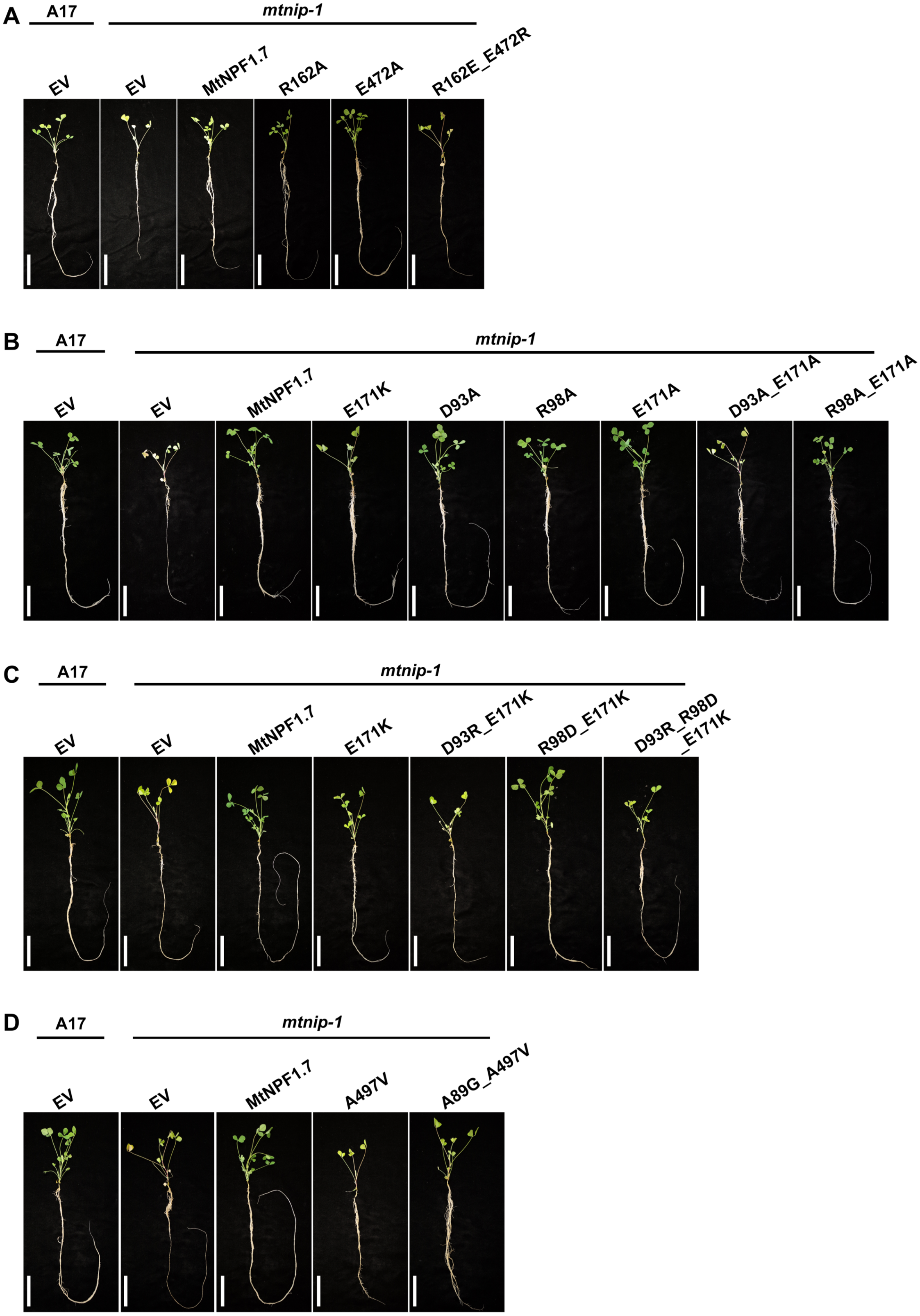
A
A17
mtnip-1
EV
EV
MtNPF1.7
R162A
E472A
R162E_E472R
B
A17
mtnip-1
EV
EV
MtNPF1.7
E171K
D93A
R98A
E171A
D93A_E171A
R98A_E171A
C
A17
mtnip-1
EV
EV
MtNPF1.7
E171K
D93R_E171K
R98D_E171K
D93R_R98D _E171K
D
A17
mtnip-1
EV
EV
MtNPF1.7
A497V
A89G_A497V

Supplementary Figure S6. Additional images of hairy root transformed *M. truncatula* plants used in this study. (A) A17 plant roots expressing the empty vector (MU06) and *mtnip-1* plant roots with expression of empty vector (MU06), MtNPF1.7_wt (pYCY400), _R162A (pYCY407), _E472A (pYCY408), and _R162E-E472R (pYCY411). **(B)** A17 plant roots transformed with empty vector (MU06) and *mtnip-1* plant roots transformed with empty vector (MU06), MtNPF1.7_wt, _E171K (pYCY402), _D93A (pYCY412), _R98A (pYCY413), _E171A (pYCY414), _D93A-E171A (pYCY415), and _R98A-E171A (pYCY416). **(C)** A17 plant roots transformed with empty vector (MU06) and *mtnip-1* plant roots transformed with empty vector (MU06), MtNPF1.7_wt, _E171K, _D93R-E171K (pYCY417), _R98D-E171K (pYCY418), and _D93R-R98D-E171K (pYCY419). **(D)** A17 plant roots transformed with the empty vector, *mtnip-1* plant roots transformed with the empty vector, MtNPF1.7_wt, _A497V (pYCY401), and A89G_A497V (pYCY420). Note that plants grouped together were grown together in the same aeroponics chamber with their respective positive and negative control plants to assure validity of data.

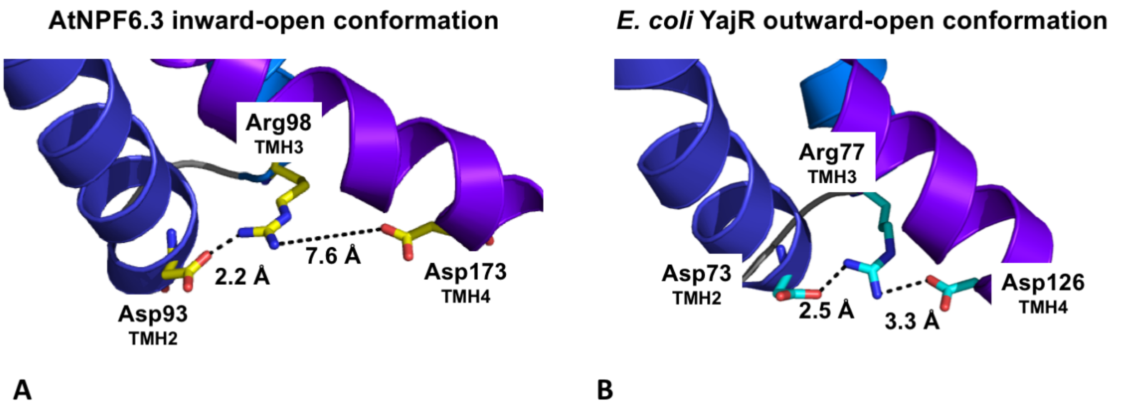

Supplementary Figure S7. Comparison of the salt bridge triad captured in two different conformations. Comparison between the salt bridge triad from **(A)** the crystal structure of AtNPF6.3 transporter solved in the inward open (I_o) conformation (pdb: 4oh3) (Sun et al. 2014) and **(B)** from the *E. coli* YajR transporter solved in the outward open conformation (O_o) (pdb: 3wdo) (Jiang et al., 2013). **(A)** AtNPF6.3 captured in the I_o conformation with Arg98 forming a salt bridge with Asp93, but Asp173 is too distant for being in a salt bridge at the same time. **(B)** YajR in the O_o conformation, with Arg77 forming a salt bridge with both Asp73 and Asp126, thanks to the close packing of the TMHs on the cytoplasmic side.

Supplemental references

Boggavarapu, R., Jeckelmann, J.M., Harder, D., Ucurum, Z., and Fotiadis, D. (2015). Role of electrostatic interactions for ligand recognition and specificity of peptide transporters. *BMC Biol.* 13, 58. doi: 10.1186/s12915-015-0167-8

Guettou, F., Quistgaard, E.M., Trésaugues, L., Moberg, P., Jegerschöld, C., Zhu, L., Jong, A.J., Nordlund, P., and Löw, C. (2013). Structural insights into substrate recognition in proton-dependent oligopeptide transporters. *EMBO Rep.* 14, 804-810. doi: 10.1038/embor.2013.107

Guettou, F., Quistgaard, E.M., Raba, M., Moberg, P., Löw, C., and Nordlund, P. (2014). Selectivity mechanism of a bacterial homolog of the human drug-peptide transporters PepT1 and PepT2. *Nat. Struct. Mol. Biol.* 21, 728-731. doi: 10.1038/nsmb.2860

Huang, C. Y., Olieric, V., Ma, P., Panepucci, E., Diederichs, K., Wang, M., and Caffrey, M. (2015). *In meso in situ* serial X-ray crystallography of soluble and membrane proteins. *Acta Crystallogr. D Biol. Crystallogr.* 71, 1238-1256. doi:10.1107/S1399004715005210

Huang, C. Y., Olieric, V., Ma, P., Howe, N., Vogeley, L., Liu, X., Warshamanage, R., Weinert, T., Panepucci, E., Kobilka, B., Diederichs, K., Wang, M., and Caffrey, M. (2016). *In meso in situ* serial X-ray crystallography of soluble and membrane proteins at cryogenic temperatures. *Acta Crystallogr. D Struct. Biol.* 72, 93-112. doi:10.1107/S2059798315021683

Lyons, J.A., Parker, J.L., Solcan, N., Brinth, A., Li, D., Shah, S.T., Caffrey, M., and Newstead, S. (2014). Structural basis for polyspecificity in the POT family of proton-coupled oligopeptide transporters. *EMBO Rep.* 15, 886-893. doi: 10.15252/embr.201338403

Martinez Molledo, M., Quistgaard, E.M., Flayhan, A., Pieprzyk, J., and Löw, C. (2018a). Multispecific substrate recognition in a proton-dependent oligopeptide transporter. *Structure* 26, 467-476. doi: 10.1016/j.str.2018.01.005

Martinez Molledo, M., Quistgaard, E.M., and Löw, C. (2018b). Tripeptide binding in a proton-dependent oligopeptide transporter. *FEBS Lett.* 592, 3239-3247. doi: 10.1002/1873-3468.13246

Nagarathinam, K., Nakada-Nakura, Y., Parthier, C., Terada, T., Juge, N., Jaenecke, F., Liu, K., Hotta, Y., Miyaji, T., Omote, H., Iwata, S., Nomura, N., Stubbs, M.T., and Tanabe, M. (2018). Outward open conformation of a Major Facilitator Superfamily multidrug/H(+) antiporter provides insights into switching mechanism. *Nat. Commun.* 9, 4005. doi: 10.1038/s41467-018-06306-x

Front. Plant Sci. | doi: 10.3389/fpls.2021.685334

Quistgaard, E.M., Martinez Molledo, M., and Löw, C. (2017). Structure determination of a major facilitator peptide transporter: inward facing PepTSt from *Streptococcus thermophilus* crystallized in space group P3121. *PLoS One*. 12(3):e0173126. doi:10.1371/journal.pone.0173126
